# Nuclear PI3P produced by the Beclin-1/Vps34 complex regulates DNA mismatch repair

**DOI:** 10.1093/nar/gkag696

**Published:** 2026-07-14

**Authors:** Xinyi Li, Mariella Vicinanza, Ana Lopez, Beatrice Paola Festa, Lars Schlotawa, Antonio Daniel Barbosa, Michael Takla, Gabriel Balmus, Angeleen Fleming, David C Rubinsztein

**Affiliations:** Cambridge Institute for Medical Research (CIMR), Department of Genomic Medicine, University of Cambridge, Cambridge CB2 0XY, United Kingdom; UK Dementia Research Institute, Cambridge Institute for Medical Research (CIMR), University of Cambridge, Cambridge CB2 0XY, United Kingdom; Cambridge Institute for Medical Research (CIMR), Department of Genomic Medicine, University of Cambridge, Cambridge CB2 0XY, United Kingdom; Cambridge Institute for Medical Research (CIMR), Department of Genomic Medicine, University of Cambridge, Cambridge CB2 0XY, United Kingdom; UK Dementia Research Institute, Cambridge Institute for Medical Research (CIMR), University of Cambridge, Cambridge CB2 0XY, United Kingdom; Department of Physiology, Development and Neuroscience, University of Cambridge, Cambridge CB2 3DY, United Kingdom; Cambridge Institute for Medical Research (CIMR), Department of Genomic Medicine, University of Cambridge, Cambridge CB2 0XY, United Kingdom; UK Dementia Research Institute, Cambridge Institute for Medical Research (CIMR), University of Cambridge, Cambridge CB2 0XY, United Kingdom; Cambridge Institute for Medical Research (CIMR), Department of Genomic Medicine, University of Cambridge, Cambridge CB2 0XY, United Kingdom; Department of Physiology, Development and Neuroscience, University of Cambridge, Cambridge CB2 3DY, United Kingdom; Cambridge Institute for Medical Research (CIMR), Department of Genomic Medicine, University of Cambridge, Cambridge CB2 0XY, United Kingdom; UK Dementia Research Institute, Cambridge Institute for Medical Research (CIMR), University of Cambridge, Cambridge CB2 0XY, United Kingdom; Cambridge Institute for Medical Research (CIMR), Department of Genomic Medicine, University of Cambridge, Cambridge CB2 0XY, United Kingdom; UK Dementia Research Institute, Cambridge Institute for Medical Research (CIMR), University of Cambridge, Cambridge CB2 0XY, United Kingdom; UK Dementia Research Institute at University of Cambridge, Department of Clinical Neurosciences, University of Cambridge, Cambridge CB2 0AH, United Kingdom; Department of Molecular Neuroscience, Transylvanian Institute of Neuroscience, Cluj-Napoca 400191, Romania; Cambridge Institute for Medical Research (CIMR), Department of Genomic Medicine, University of Cambridge, Cambridge CB2 0XY, United Kingdom; UK Dementia Research Institute, Cambridge Institute for Medical Research (CIMR), University of Cambridge, Cambridge CB2 0XY, United Kingdom; Department of Physiology, Development and Neuroscience, University of Cambridge, Cambridge CB2 3DY, United Kingdom; Cambridge Institute for Medical Research (CIMR), Department of Genomic Medicine, University of Cambridge, Cambridge CB2 0XY, United Kingdom; UK Dementia Research Institute, Cambridge Institute for Medical Research (CIMR), University of Cambridge, Cambridge CB2 0XY, United Kingdom

## Abstract

Genome integrity relies on DNA mismatch repair (MMR) to correct replication errors, yet whether non-protein cofactors regulate this pathway remains unexplored. Here, we identify nuclear phosphatidylinositol-3-phosphate (PI3P) as a lipid regulator of MMR. Using biosensors, lipid pulldown, and proximity ligation assays, we show that PI3P forms discrete nuclear puncta in close proximity to the MutSα (MSH2:MSH6) and MutSβ (MSH2:MSH3) MMR recognition complexes. Pharmacological or genetic depletion of the class III PI3-kinase Vps34 impaired MutSα and MutSβ heterodimer assembly without altering MMR protein nuclear abundance, compromised DNA substrate association of MMR components in nuclear extracts, and elevated microsatellite instability at mononucleotide repeats. Exogenous PI3P enhanced MMR recognition complex assembly and DNA association in PI3P-deficient nuclear extracts, supporting a role for PI3P in promoting MMR. We further show that a nuclear Beclin-1/Vps34 complex produces this PI3P pool through an autophagy-independent mechanism. Functionally, loss of nuclear PI3P blunts MMR-dependent DNA damage signaling and confers 6-thioguanine resistance in cultured cells and in Beclin-1-deficient zebrafish *in vivo*. These findings reveal an autophagy-independent nuclear function for the Beclin-1/Vps34 complex in genome maintenance and identify PI3P as a lipid mediator of MMR, thereby expanding the functional repertoire of nuclear phosphoinositide signaling.

## Graphical Abstract

**Graphical Abstract:**
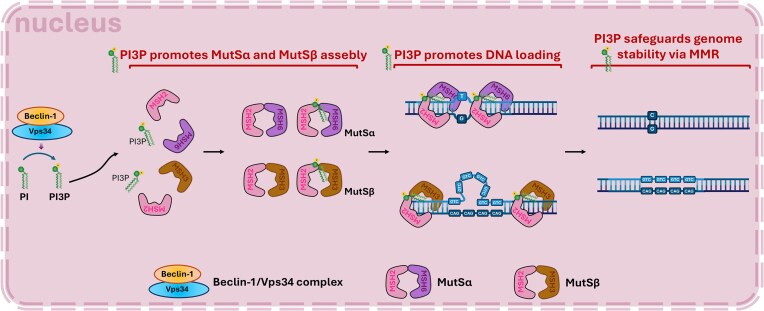

## Introduction

Genome stability is essential for cellular viability and tumor suppression, yet it is persistently challenged by endogenous replication errors and diverse genotoxic stressors [1–3]. To preserve genomic integrity, cells rely on an integrated network of DNA damage response pathways that detect, signal, and repair lesions throughout the cell cycle [4–7]. Among these pathways, DNA mismatch repair (MMR) plays a unique and indispensable role by correcting base–base mismatches and insertion–deletion loops generated during DNA replication [8–13]. The MMR process is initiated by lesion recognition through the MutSα (MSH2:MSH6) or MutSβ (MSH2:MSH3) recognition complexes, which subsequently recruit members of the MutL family, most prominently MutLα (MLH1:PMS2), to facilitate the coordination of downstream repair events involving exonuclease 1 (EXO1) and DNA polymerase δ (Pol δ) for strand excision and resynthesis [9, 14]. Defects in MMR lead to microsatellite instability (MSI), hypermutation, and predisposition to cancers such as Lynch syndrome [15–18] and strongly influence cellular responses to chemotherapeutics whose cytotoxicity depends on functional MMR, including thiopurines and alkylating agents [19–22]. Despite extensive characterization of the protein factors that drive MMR, the contribution of non-protein regulators has remained largely unexplored.

One emerging area of interest is nuclear phosphoinositide signaling. Phosphoinositides are classically viewed as cytoplasmic lipids that define membrane identity and coordinate autophagic and endosomal dynamics [23–25]. Phosphatidylinositol-3-phosphate (PI3P), in particular, is canonically produced by the class III phosphatidylinositol 3-kinase Vps34 in complex with Beclin-1 and is required for autophagosome biogenesis and endosomal trafficking [25, 26]. For decades, PI3P has been considered a lipid confined to membrane-bound organelles [27, 28]. However, accumulating evidence indicates that phosphoinositides, including PI3P, also operate within the nucleus [29–32], and recent studies have reported nuclear relocalization of Beclin-1/Vps34 complex components with suggested autophagy-independent functions [33–37]. The physiological relevance and molecular targets of nuclear PI3P, however, remain poorly defined.

Here, we investigate the role of nuclear PI3P, the canonical lipid product of the Beclin-1/Vps34 complex, in regulating MMR. We show that PI3P is present in the nucleus and is associated with the core MMR machinery. PI3P depletion through Vps34 pharmacological inhibition or genetic perturbation impaired MutSα and MutSβ assembly, reduced DNA substrate association of MMR components in a *pseudo-in vitro* DNA-binding assay using nuclear extracts, increased microsatellite instability at mononucleotide repeats, and blunted MMR-dependent DNA damage signaling governing cell fate. Supplementation of exogenous PI3P in PI3P-deficient nuclear extracts enhanced MMR recognition complex assembly and DNA association, supporting a role for PI3P in promoting MMR. We further show that this nuclear PI3P pool is generated by the nuclear Beclin-1/Vps34 complex. Disruption of this complex reduced nuclear PI3P production, compromised MMR recognition complex assembly and DNA association, impaired MMR function, and conferred a survival advantage to both cultured cells and zebrafish exposed to 6-thioguanine. Collectively, our findings position nuclear PI3P as a lipid regulator of MMR, reveal a previously unrecognized autophagy-independent role of the Beclin-1/Vps34 complex, and highlight the broader relevance of nuclear phosphoinositide signaling in safeguarding genome integrity.

## Materials and methods

### Cell culture

HeLa BECN1 knockout (BECN1^−/−^) cells generated by TALEN and their matched wild-type (WT) controls were a gift from Wensheng Wei (Peking University, Beijing) [38]. HeLa, WT and BECN1^−/−^ cells were maintained in Dulbecco’s modified Eagle’s medium (DMEM, Merck D6429) supplemented with 10% (v/v) fetal bovine serum (FBS, Sigma–Aldrich) at 37°C in a humidified incubator with 5% CO₂. Cultures were regularly tested for Mycoplasma contamination and were not passaged beyond standard limits for functional assays.

### Antibodies and reagents

Primary antibodies used for immunoblotting included: anti-MSH2 [mouse, 1:1000, Thermo Fisher 33-7900; rabbit, 1:1000, Cell Signaling Technology (CST) 2017], anti-MSH3 (rabbit, 1:2000, Proteintech 22393-1-AP), anti-MSH6 (rabbit, 1:1000, Abcam ab92471), anti-PMS2 (mouse, 1:1000, Santa Cruz sc-25315), anti-MLH1 (rabbit, 1:1000, Abcam ab92312), anti-VPS34 (rabbit, 1:1000, Abcam ab227861), anti-Beclin-1 (rabbit, 1:1000, CST 3738), anti-GFP (mouse, 1:5000, Proteintech 66002-1-Ig), anti-mCherry (mouse, 1:5000, Proteintech 68088-1-Ig), anti-γH2AX (rabbit, 1:1000, CST 2577), anti-LC3 (rabbit, 1:1000, Proteintech 14600-1-AP), anti-α-Tubulin (mouse, 1:3000, CST 3873), anti-Lamin B1 (mouse, 1:3000, Proteintech 66095-1-Ig), and anti-GAPDH (mouse, 1:3000, Abcam ab8245). DyLight-conjugated anti-rabbit 800 and anti-mouse 680 secondary antibodies (Thermo Fisher SA5-10036 and 35518) were used at 1:2000. Anti-FLAG antibody (rabbit, Cell Signaling #14793S) was used at 1:1000 for staining cells for fluorescence-activated cell sorting, together with Alexa Fluor^®^ 647 Conjugate (Cell Signaling #4414).

Primary antibodies for immunofluorescence (IF) and proximity ligation assays (PLA) included: anti-γH2AX (rabbit, 1:400, CST 2577), anti-MSH2 (mouse, 1:400, Thermo Fisher 33-7900; rabbit, 1:400, CST 2017), anti-MSH3 (rabbit, 1:200, Abcam ab111107), anti-MSH6 (rabbit, 1:400, Abcam ab208940), anti-PI3P (mouse, 1:200, Echelon Z-P003), and anti-FLAG (mouse, 1:800, Sigma F1804). Alexa Fluor-conjugated goat anti-mouse and anti-rabbit secondary antibodies (Thermo Fisher Scientific) were used at 1:600 for IF.

Cells were treated with the following compounds at the indicated concentrations and durations unless otherwise specified in figure legends: 6-thioguanine (6-TG, 30 µM, 3 h, harvested 24 h after treatment, Sigma–Aldrich A4882), tert-butyl hydroperoxide (TBHP, 100 µM, 45 min, Merck 458 139), SAR405 (2 µM, 16 h, APExBIO A8883), IN1 (1 µM, 16 h, Selleckchem S7980), and bafilomycin A1 (400 nM, 4 h, Enzo LifeSciences BML-CM110). DMSO (Merck D8418) was used as vehicle control.

### DNA constructs and RNA interference

Beclin-1 expression constructs were cloned into pCMV-5a with a C-terminal FLAG tag: pCMV-5a-FLAG (empty vector), pCMV-5a-WT Beclin-1-FLAG (WT Beclin-1), and pCMV-5a-Beclin-1 L184A/L187A-NLS-FLAG (nuclear Beclin-1). BECN1 was additionally silenced using shRNA constructs (sh_BECN1_1 RHS4430-200242020, sh_BECN1_2 RHS4430-200244518; non-targeting shRNA RHS4348).

The dual-fluorescence MSI reporter constructs mCherry-N23-P2A-EGFP (N23 control) and mCherry-G23-P2A-EGFP (G23 MSI reporter) were synthesized by Genewiz in a pEGFP-N1 backbone. The GFP-2×FYVE PI3P biosensor was a kind gift from Dr Claudia Puri.

Plasmid DNA and shRNA vectors were transfected using TransIT-2020 (Mirus MIR5400) according to the manufacturer’s instructions. Pre-designed siRNAs (Dharmacon) were used to deplete VPS34 (5ʹ-GAAGUUCUCAGGACUAUAU-3ʹ) and MSH2 (5ʹ-UCUGCAGAGUGUUGUGCUU-3ʹ), with a non-targeting siRNA (D-001810-10) as control. siRNAs were delivered using Lipofectamine 2000 (Invitrogen 11668019).

### Immunofluorescence and proximity ligation assay

For IF and PLA, cells were seeded on glass coverslips (confocal imaging) or in 96-well optical-bottom plates (high-content imaging). Cells were fixed in 2%–4% paraformaldehyde for 5 min at room temperature (RT), washed with PBS, and permeabilized with 0.5% Triton X-100 in PBS for 8 min. After blocking with 1% (w/v) BSA in PBS containing 0.1% Triton X-100 (PBST) for 1 h at RT, cells were incubated with primary antibodies diluted in 1% BSA–PBST overnight at 4°C. For detection of overexpressed Beclin-1 constructs, anti-FLAG antibody was used. Following three 5-min washes with PBST, cells were incubated with Alexa Fluor-conjugated secondary antibodies for 1 h at RT, washed, and mounted with ProLong Gold Antifade containing DAPI (Thermo Fisher, P36935). For plate-based assays, cells were stained with DAPI (Merck, 9542), washed, and stored in PBST until imaging.

For PI3P visualization, cells were either transfected with GFP-2×FYVE overnight, then fixed and processed as above, or incubated with anti-PI3P primary antibody.

PLA was performed using the Duolink Proximity Ligation Kit (Sigma–Aldrich) according to the manufacturer’s instructions, using validated antibody pairs. Negative controls included omission of primary antibodies or use of single primaries only.

Confocal imaging was performed on a Zeiss LSM880 microscope using a 63× oil-immersion objective with ZEN Black software. High-content imaging was performed on a Thermo CellInsight CX7 using a 40× air objective and HCS Studio 5.0. Nuclear intensity of γH2AX foci, PLA puncta, and PI3P puncta were quantified using Fiji (ImageJ) and HCS Studio 5.0.

### Nuclear fractionation

For nuclear fractionation prior to immunoprecipitation (IP) or PI3P pulldown, cells were washed with ice-cold PBS and incubated in hypotonic nuclear fractionation buffer (10 mM HEPES pH 7.5, 10 mM KCl, 1.5 mM MgCl₂, 34 mM sucrose, 10% w/w glycerol, 0.1% w/w Triton X-100, freshly supplemented with 1 mM DTT and 1× protease inhibitor cocktail) for 5 min at 4°C on a rotator. Crude nuclei were pelleted at 1300 × *g* for 4 min at 4°C and washed two to three times in the same buffer to remove cytoplasmic contaminants.

### Immunoprecipitation in nuclear extracts

To assess MutSα and MutSβ complex formation, IPs were performed from nuclear lysates. Washed nuclei were lysed in IP lysis buffer (150 mM NaCl, 25 mM Tris–HCl pH 7.5, 1 mM EDTA, 1% w/w NP-40, 5% w/w glycerol, freshly supplemented with protease and phosphatase inhibitors). Lysates were sonicated, incubated for 30 min at 4°C on a rotator, and cleared at 16 100 × *g* for 10 min at 4°C. Protein concentrations were determined using a BCA assay (Thermo Fisher 23225). Input samples were reserved, and the remaining lysate was incubated overnight at 4°C with rabbit anti-MSH2 (1:200, CST 2017) or rabbit IgG isotype control (CST 3900). Dynabeads Protein A (Thermo Fisher 10002D) were then added and incubated for 1 h at 4°C. Beads were collected on a magnetic rack, washed five times with IP lysis buffer, and bound proteins were eluted in Laemmli sample buffer, boiled for 10 min at 95°C, and analyzed by immunoblotting or mass spectrometry.

### PI3P pulldown in nuclear extracts

To identify nuclear PI3P-interacting proteins, PI3P pulldown was performed from nuclear extracts. Nuclei were lysed in PI3P pulldown buffer (150 mM NaCl, 10 mM HEPES pH 7.5, 0.25% w/w NP-40, freshly supplemented with protease and phosphatase inhibitors), sonicated, and cleared at 16 100 × *g* for 10 min at 4°C. Nuclear lysates were incubated with control beads (Echelon P-B000) or PI-conjugated beads (Echelon P-B00S), including PI3P-conjugated beads (Echelon P-B003A), for 4 h at 4°C on a rotator. Beads were collected at 800 × *g* for 3 min at 4°C, washed five times with lysis buffer, and bound proteins were eluted in Laemmli buffer, boiled for 10 min at 95°C, and analyzed by immunoblotting or mass spectrometry.

### Western blotting

Cells were washed with PBS and lysed in RIPA buffer (50 mM Tris–HCl pH 8.0, 40 mM NaCl, 2 mM MgCl₂, 0.5% Triton X-100) freshly supplemented with benzonase, protease, and phosphatase inhibitor cocktails. Protein concentrations were determined using a BCA assay. Equal amounts of protein were mixed with Laemmli buffer, boiled for 10 min at 95°C, resolved on 8%, 10%, or 15% sodium dodecyl sulfate–polyacrylamide gel electrophoresis, and transferred to methanol-activated PVDF membranes. Membranes were blocked in 5% (w/v) skimmed milk in TBS with 0.1% Tween-20 (TBST) for 1 h at RT, incubated overnight at 4°C with primary antibodies in 5% BSA/TBS, washed three times in TBST, and incubated with DyLight-conjugated secondary antibodies (1:2000 in 5% BSA/TBST) for 1 h at RT. After three additional TBST washes, blots were imaged on an Odyssey Infrared Imaging System (LI-COR). Band intensities were quantified using Image Studio Lite, and protein levels were normalized to the indicated loading controls.

### Mass spectrometry analysis

Mass spectrometry data from PI3P and Beclin1 pulldown experiments were normalized to control samples using a pseudocount transformation to stabilize variance. Specifically, intensity values were divided by the average control intensity plus a pseudocount, followed by log_2_ transformation to yield fold-change scores. Proteins with a ≥16-fold enrichment (log_2_ > 4) relative to control were considered significant. A stringent inclusion filter was applied to retain proteins reproducibly detected across replicates and above background noise. These filtered, high-confidence interactors were ranked by enrichment and retained for overlap analysis between PI3P and Beclin1 datasets. The resulting shared interactome formed the input for STRING network modeling and subsequent functional clustering. Proteins enriched in both PI3P and Beclin1 pulldowns were intersected to define a shared interactome, which was submitted to STRING (v12) using high-confidence interaction scores (minimum 0.700). The resulting network included both experimentally validated and predicted interactions, with evidence from curated databases, co-expression, and physical interactions. The STRING network was imported into Cytoscape (v3.9.1) for visualization and cluster annotation. Clustering was performed using the Markov Cluster Algorithm (MCL) with an inflation parameter of 2.0. KEGG pathway enrichment was performed directly within Cytoscape using the stringApp plugin, with a false discovery rate (FDR) threshold of 0.01. String analysis was done using version-12-0.string-db.org on 30 January 2026. The original proteomics data have been deposited to the ProteomeXchange Consortium via the PRIDE partner repository with accession code PXD066473. The processed proteomics data for analysis and illustration is provided in Supplementary Table S1.

### *Pseudo*-*in vitro* DNA binding assay

- Nuclear extract preparation

For *pseudo-in vitro* DNA binding assays, nuclear extracts were prepared using a high-salt protocol. Cells were washed with ice-cold PBS and resuspended in hypotonic buffer (10 mM HEPES, 1.5 mM MgCl₂, 10 mM KCl, freshly supplemented with 0.5 mM DTT and protease/phosphatase inhibitors). Cells were sheared by passage through a 25-gauge needle and incubated for 20 min at 4°C on a rotator. Nuclei were pelleted at 2000 × *g* for 10 min at 4°C, washed three times with hypotonic buffer, and then pelleted at 25 000 × *g* for 2 min at 4°C. Nuclear pellets were lysed in hypertonic buffer (20 mM HEPES, 1.5 mM MgCl₂, 420 mM NaCl, 0.25 mM EDTA, 25% w/w glycerol, freshly supplemented with 0.5 mM DTT and protease/phosphatase inhibitors) using a 28-gauge needle. Lysates were sonicated, incubated for 30 min at 4°C on a rotator, and cleared at 25 000 × *g* for 10 min at 4°C. Supernatants were dialyzed overnight at 4°C against dialysis buffer (20 mM HEPES, 0.2 mM EDTA, 0.5 mM DTT, protease/phosphatase inhibitors), re-clarified at 25 000 × *g* for 10 min, and snap-frozen in liquid nitrogen. Protein content was assessed by Coomassie staining.

- DNA substrates and bead coupling

Unlabeled and biotin-labeled 114-nt oligonucleotides were obtained from Sigma–Aldrich. Oligos were annealed in NEBuffer 3.1 by heating to 95°C followed by slow cooling to 91.7°C (homoduplex: 114G-bio/114G with 114C-homo) or 91.2°C (heteroduplex: 114G-bio/114G with 114T-hetero), repeating the heating/cooling cycle three times, then slowly cooling to 4°C. Annealing efficiency was verified on 2.5% agarose gels and double-stranded DNA (dsDNA) concentrations were determined by NanoDrop. Biotin-labeled dsDNA was coupled to streptavidin beads (Thermo Fisher 65001) in coupling buffer (5 mM Tris pH 7.4, 0.5 mM EDTA, 1 M NaCl) by rotation for 45 min at RT.

- DNA binding reactions

DNA-coupled beads were washed in bead buffer (20 mM Tris pH 7.9, 50 mM NaCl, 5% w/w glycerol, 1 mM EDTA) and incubated with nuclear extracts in reaction buffer (20 mM Tris pH 7.9, 50 mM NaCl, 1.5 mM MgCl₂, 5% w/w glycerol, 1 mM EDTA, 0.5 mM DTT, 3 µg/ml poly[d(I–C)], plus protease/phosphatase inhibitors) for 30 min at 4°C on a rotator. Where indicated, PI (Echelon PIP diC8, *P*-0008) or PI(3)P [Echelon PI(3)P diC8, *P*-3008] was added to a final concentration of 10 µM. Beads were collected on a magnetic rack, washed five times with reaction buffer, and bound proteins were eluted with Laemmli buffer, boiled for 10 min at 95°C, and analyzed by immunoblotting.

### Dual-fluorescence MSI reporter and flow cytometry

WT and BECN1^−/−^ HeLa cells were transfected with the G23 MSI reporter or N23 control using TransIT-2020. After 7 days, mCherry-positive cells were isolated by flow sorting, expanded briefly, and cryopreserved as assay stocks. For experiments, thawed cells were allowed to recover, treated with drugs or siRNAs as indicated, cultured for 1 week, and harvested for flow cytometry.

Cells were washed with PBS, detached with trypsin, filtered through 40-µm strainers, and kept on ice during acquisition. Data were collected on an Attune NxT flow cytometer (Thermo Fisher Scientific). Non-fluorescent cells served as negative controls for gating. Data were analyzed with FlowJo™ 10 (TreeStar), and MSI was quantified as the mCherry^+^EGFP^+^/mCherry^+^ fraction.

### Clonogenic survival assays

For clonogenic assays, 800 cells per well were seeded in six-well plates (Day 0). Medium was replaced on Days 1, 3, 5, 7, 9, and 11 with vehicle or 6-TG at the indicated concentrations. For assays with Vps34 inhibition, Day 1 medium contained 1 µM IN1 or 2 µM SAR405 together with 6-TG; from Day 2 onward, inhibitor concentrations were reduced to 100 nM IN1 or 200 nM SAR405 and maintained with 6-TG. On Day 13, all wells were switched to drug-free medium. On Day 14, colonies were fixed and stained with crystal violet solution (4% w/w paraformaldehyde, 0.05% w/w crystal violet) for 10 min at RT, washed with water, air-dried, and counted using Fiji (ImageJ). For each condition, at least three technical replicates were included per biological replicate.

### Zebrafish husbandry and genetic manipulation

All zebrafish experiments were conducted in accordance with the UK Animals (Scientific Procedures) Act, under approved Home Office Project and Personal License and with University of Cambridge Animal Welfare and Ethical Review Body approval. Experiments followed PREPARE and ARRIVE guidelines. Zebrafish were maintained on a 14-h light/10-h dark cycle at 28.5°C in standard conditions. Embryos were collected from natural spawning, staged according to established criteria, and reared in embryo medium (5 mM NaCl, 0.17 mM KCl, 0.33 mM CaCl₂, 0.33 mM MgSO₄, 5 mM HEPES pH 7.2). The pool of embryos collected from a single cross at the same time is defined as a clutch, equivalent to a biological replicate.

The heterozygous *becn1* mutant line (*becn1*^sa14704^) was obtained from the Zebrafish Mutation Project [39]. The line harbors a nonsense (G/T) mutation in exon 6 of 12 of *becn1*, resulting in a premature stop codon at amino acid 154 (of 447). Incrosses of heterozygous adults were used to generate WT (*becn1*^+/+^), heterozygous (*becn1*^+/−^), and homozygous null (*becn1*^−/−^) offspring, which were genotyped by allele-specific amplification using a custom-designed KASP assay from LGC Genomics.

For CRISPR-mediated becn1 disruption in WT background, a guide RNA targeting beclin-1 (UGUGGUGUAUUCACCUCAGUUUUAG) was designed using the Dharmacon CRISPR tool. CRISPR guide RNA, tracrRNA (Dharmacon Edit-R), and Cas9 nuclease protein NLS (Horizon CAS12206) were complexed as described previously. Approximately 4.28 nL of CRISPR–Cas9 mix was injected into the yolk of one-cell-stage embryos from TL WT fish; uninjected siblings served as controls.

### Genotoxicity assays in zebrafish

The toxicity of the genotoxic agents 6-TG was evaluated by adding these compounds to the embryo medium at 1 d.p.f. and monitoring larvae daily for any adverse effect. Final concentrations of 600 μM 6-TG were maintained from 1 to 5 d.p.f. by replenishing drugs and medium daily. Fish with morphological abnormalities such as shorter bodies or pericardial edemas were counted in all groups blindly (see Fig. 6F for examples). Toxicity was then represented as the percentage of unhealthy fish in each group. Drug treatment using the *beclin1^sa14704^* mutant line was performed in 3 independent clutches with a minimum of 40 fish each, whereas the toxicity assays in Beclin1 CRISPR-injected TL WT fish *versus* their uninjected siblings were quantified in 7 independent clutches with a minimum of 100 fish each.

### Statistical analysis

- Data acquisition and quantification of experimental readouts

Immunoblot band intensities were quantified using Image Studio Lite (Version 6.0.0). For total protein measurements, signals were first normalized to the indicated loading control, typically Lamin B1 for nuclear fractions. For co-immunoprecipitation experiments, band intensities of co-immunoprecipitated proteins were normalized to the amount of immunoprecipitated bait protein, and data are presented as the indicated co-IP/IP ratios (for example, MSH6/MSH2 and MSH3/MSH2) to estimate MutS complex assembly. For *pseudo-in vitro* DNA-binding assays, signals from bead-bound proteins were quantified and normalized to the corresponding input signal for each protein, and DNA-binding-associated readouts are presented as bead-bound/input ratios. Nuclear PLA, γH2Ax, and PI3P puncta intensity were all calculated using Fiji (ImageJ) and HCS Studio 5.0. For MSI assays with flow cytometry, MSI was quantified by flow cytometry as the mCherry^+^EGFP^+^/mCherry^+^ fraction. Where applicable, analysis was restricted to the indicated transfected population and quantified as FLAG^+^mCherry^+^EGFP^+^/FLAG^+^mCherry^+^.

- Data normalization and statistical analysis

Unless otherwise indicated in the figure legends, quantitative values were normalized within each biological replicate to the mean of all matched conditions included in that comparison set for the corresponding experiment. For example, for a comparison among conditions A, B, C, and D within a given replicate, the normalized values were calculated as A/mean(A,B,C,D), B/mean(A,B,C,D), C/mean(A,B,C,D), and D/mean(A,B,C,D). Statistical analyses were performed on these normalized biological replicate values in GraphPad Prism 10. Values are presented as mean ± SEM. Statistical comparisons were performed using paired, two-tailed Student’s t-tests on normalized biological replicate means, unless otherwise specified. At least three biological independent experiments were performed for each quantified assay. Exact n values and statistical tests are indicated in the figure legends. Significance thresholds are denoted as *P < .05, **P < .01, ***P < .001, ****P < .0001.

## Results

### Nuclear PI3P associates with DNA MMR machinery

**Figure 1.**
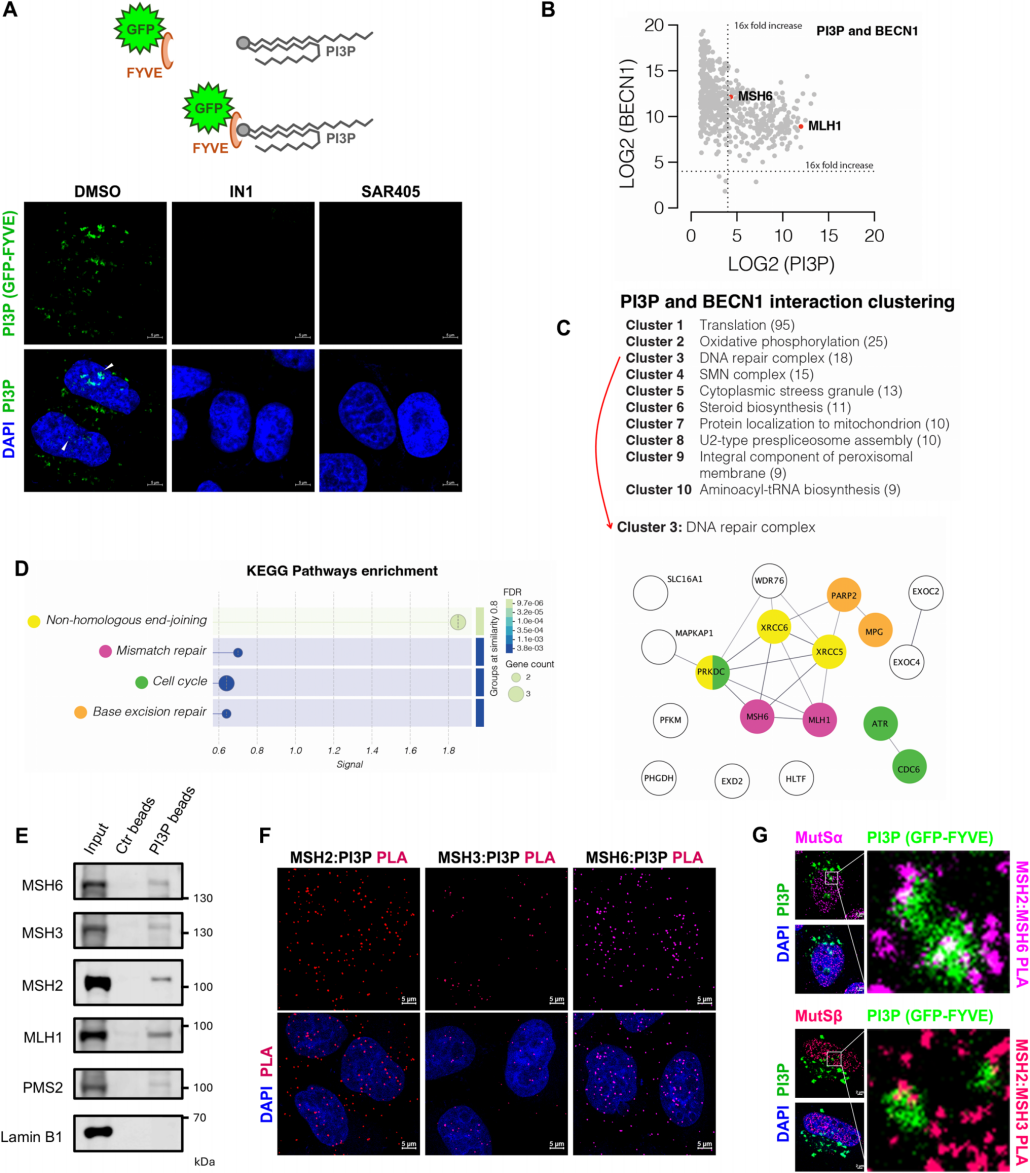
Nuclear PI3P associates with MMR machinery. (**A**) Representative confocal images of HeLa cells expressing the GFP-2×FYVE PI3P biosensor following treatment with vehicle control (DMSO) or Vps34 inhibitors IN1 (1 μM) or SAR405 (2 μM) for 16 h. White arrowheads indicate nuclear PI3P puncta. Scale bar: 5 μm. (**B**) Shared interactors between PI3P and Beclin1 pulldowns in the nucleus, with MSH6 and MLH1 highlighted. (**C**) Shared interactors between PI3P and Beclin-1 in the nucleus were clustered into ten functional groups (via STRING). (**D**) Cluster 3 was annotated as a DNA repair complex containing NHEJ proteins (yellow), MMR proteins (pink), base excision repair factors (orange), and cell cycle regulators (green). KEGG pathway enrichment of this cluster (left) reveals statistically significant enrichment for multiple DNA repair pathways (FDR < 0.01). (**E**) Immunoblot analysis of PI3P pulldown from HeLa nuclear extracts. PI3P-coated beads specifically recovered MSH2, MSH3, MSH6, MLH1, and PMS2, whereas control phosphoinositide-coated beads did not. Representative of *n* = 3 independent experiments. (**F**) Proximity ligation assay (PLA) detecting association between PI3P and MSH2 (left), MSH3 (middle), or MSH6 (right) in HeLa cell nuclei. PLA signals appear as discrete red puncta. Nuclei were counterstained with DAPI. Scale bar: 5 μm. Antibody specificity controls are shown in Supplementary Fig. S1. Representative of *n* = 3 independent experiments. (**G**) Colocalization of GFP-2×FYVE-labeled PI3P puncta with PLA signals marking MutSα (MSH2:MSH6; top; in magenta) or MutSβ (MSH2:MSH3; bottom; in red) heterodimers in HeLa cell nuclei. Magnified views of boxed regions are shown on the right. Scale bar: 2 μm. Representative of *n* = 3 independent experiments.

PI3P is canonically regarded as a cytoplasmic phosphoinositide enriched on early endosomes and autophagic membranes [25, 40–42]. However, emerging evidence in previous work has suggested the presence of PI3P within the nucleus [29, 30, 32, 43]. To directly assess nuclear PI3P, we employed the GFP-2xFYVE biosensor, whose tandem FYVE domains confer high specificity for PI3P [44–46]. In HeLa cells, GFP-2×FYVE produced the expected cytoplasmic puncta and also clearly marked discrete nuclear puncta (Fig. 1A, white arrowheads). Inhibition of Vps34, the principal class III PI3-kinase responsible for PI3P synthesis, with the potent Vps34-selective inhibitors IN1 [47] or SAR405 [48] substantially reduced GFP-2×FYVE-labeled PI3P in both the cytoplasm and nucleus (Fig. 1A), indicating that nuclear PI3P presence depends on Vps34 kinase activity.

To explore the functional context of nuclear PI3P, we performed pulldown experiments from nuclear extracts using PI3P-coated beads, with uncoated beads serving as the nonspecific binding control [49]. In parallel, pulldowns using Beclin-1 as bait in nuclear extracts were also performed to define the shared interactome, improving the resolution of candidate nuclear PI3P effectors (Supplementary Table S1). Comparative enrichment analysis identified unique (Supplementary Fig. S1A) and overlapping interactors (Fig. 1B). We then focused on the shared protein subset, reasoning that these represent convergence points of nuclear PI3P–Beclin-1 signaling. This subset was submitted to STRING functional interaction analysis [50], and the resulting network was visualized in Cytoscape [51] (Fig. 1C). Clustering of the network revealed 10 functional modules, with enrichment identifying pathways including translation, oxidative phosphorylation, RNA processing, and DNA repair. These findings are consistent with known roles of PI3P and Beclin1 in coordinating autophagy, vesicular trafficking, and metabolic adaptation [52, 53]. Among these, Cluster 3 emerged as a DNA repair complex (Fig. 1D), enriched for proteins involved in non-homologous end joining (NHEJ), such as XRCC6 (Ku70), XRCC5 (Ku80), and PRKDC (DNA-PKcs), in line with established roles of Beclin1 and PI3P in DSB repair [34, 36, 54]. Unexpectedly, this cluster also contained DNA MMR proteins, represented by MSH6 and MLH1, suggesting a novel interface between lipid signaling and MMR in the nucleus.

Consistent with this enrichment profile, immunoblot analysis of PI3P pulldowns showed that MSH2, MSH3, and MSH6, the core subunits of the MutSα and MutSβ MMR recognition complexes, together with the MutLα components PMS2 and MLH1, were recovered specifically by PI3P-coated beads but not by control beads (Fig. 1E). We further expanded this analysis using a panel of phosphoinositide-conjugated beads applied to the same nuclear extract preparation. Under these matched conditions, PI3P showed the strongest enrichment for the tested MMR components, whereas only weaker recovery of some proteins, particularly MSH2 and MSH3, was observed with certain other phosphoinositide species (Supplementary Fig. S1B), suggesting preferential association of these MMR factors with PI3P and suggesting the high specificity of the interaction between PI3P and these MMR proteins. We next examined whether PI3P and MMR proteins resided in close proximity *in situ* using PLA [55]. Antibody specificity controls, including omission of primary antibodies or use of single primary antibodies, yielded no detectable PLA signal, confirming assay specificity (Supplementary Fig. S1C). Under these validated conditions, robust PLA signals were detected between PI3P and MSH2, MSH3, or MSH6 in HeLa nuclei (Fig. 1F), confirming close proximity between PI3P and MMR recognition complex components *in situ* in the nuclear compartment. Moreover, nuclear PI3P puncta, detected by the GFP-2×FYVE probe, colocalized with PLA signals marking MutSα (MSH2:MSH6) and MutSβ (MSH2:MSH3) heterodimers (Fig. 1G). Together, these data demonstrate that a pool of nuclear PI3P physically associates with the core MMR machinery, suggesting a potential regulatory role for PI3P in MMR.

### PI3P promotes MutSα and MutSβ MMR recognition complex assembly and DNA association

**Figure 2.**
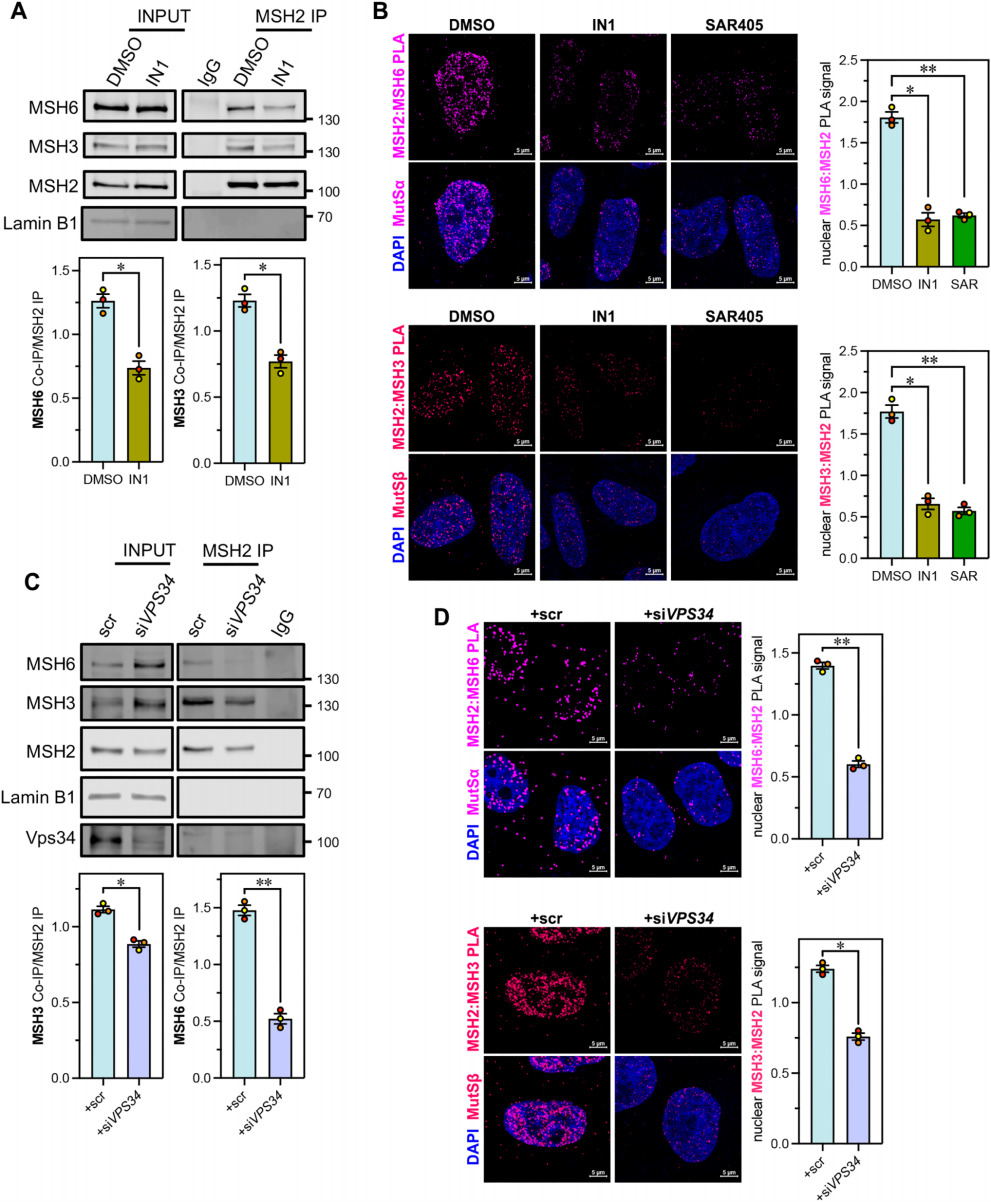
PI3P promotes MutSα and MutSβ assembly. (**A**) Immunoblot analysis of MSH2 immunoprecipitation (IP) from nuclear extracts of HeLa cells treated with DMSO or IN1 (1 μM, 16 h). Co-immunoprecipitated MSH6 and MSH3 indicate MutSα and MutSβ assembly, respectively. Quantification of MSH6/MSH2 and MSH3/MSH2 ratios is shown below. *n* = 3 biological replicates. *P < .05. (**B**) PLA detecting MutSα (MSH2:MSH6, top) and MutSβ (MSH2:MSH3, bottom) in HeLa cells treated with DMSO, IN1 (1 μM), or SAR405 (2 μM) for 16 h. Quantification of nuclear PLA foci per cell is shown on the right. Scale bar: 5 μm. *n* = 3 biological replicates. *P < .05, **P < .01. (**C**) Immunoblot analysis of MSH2 IP from nuclear extracts of HeLa cells transfected with scrambled (scr) or *VPS34*-targeting siRNA (si*VPS34*). Quantification of MSH6/MSH2 and MSH3/MSH2 ratios is shown below. *n* = 3 biological replicates. *P < .05, **P < .01. (**D**) PLA detecting MutSα (left) and MutSβ (right) in HeLa cells transfected with scr or si*VPS34*. Quantification of nuclear PLA foci per cell is shown on the right. Scale bar: 5 μm. *n* = 3 biological replicates. *P < .05, **P < .01.

Given the interactions between PI3P and MMR components, we next examined whether PI3P contributes functionally to MMR. Nuclear levels of MSH2, MSH3, and MSH6 remained unchanged following PI3P depletion with IN1 or SAR405 treatment (Supplementary Fig. S2A), indicating that PI3P does not regulate the abundance of these proteins in the nucleus. However, immunoprecipitation in nuclear extracts revealed that IN1 treatment compromised the physical interactions of MSH2 with MSH3 and MSH6 (Fig. 2A), indicative of impaired MutSα and MutSβ heterodimer formation under PI3P-deficient conditions. Consistent with this biochemical readout, PI3P depletion with IN1 or SAR405 substantially reduced the PLA signals for nuclear MutSα and MutSβ complexes (Fig. 2B). Similarly, siRNA-mediated depletion of Vps34, which did not affect nuclear abundance of MMR proteins (Supplementary Fig. S2B and C), reduced the co-immunoprecipitation of MSH6 and MSH3 with MSH2 (Fig. 2C), and decreased the PLA signals reporting nuclear MutSα and MutSβ complexes (Fig. 2D). Together, these findings indicate that Vps34-dependent PI3P production in the nucleus promotes proper formation of the MMR recognition complexes.

**Figure 3.**
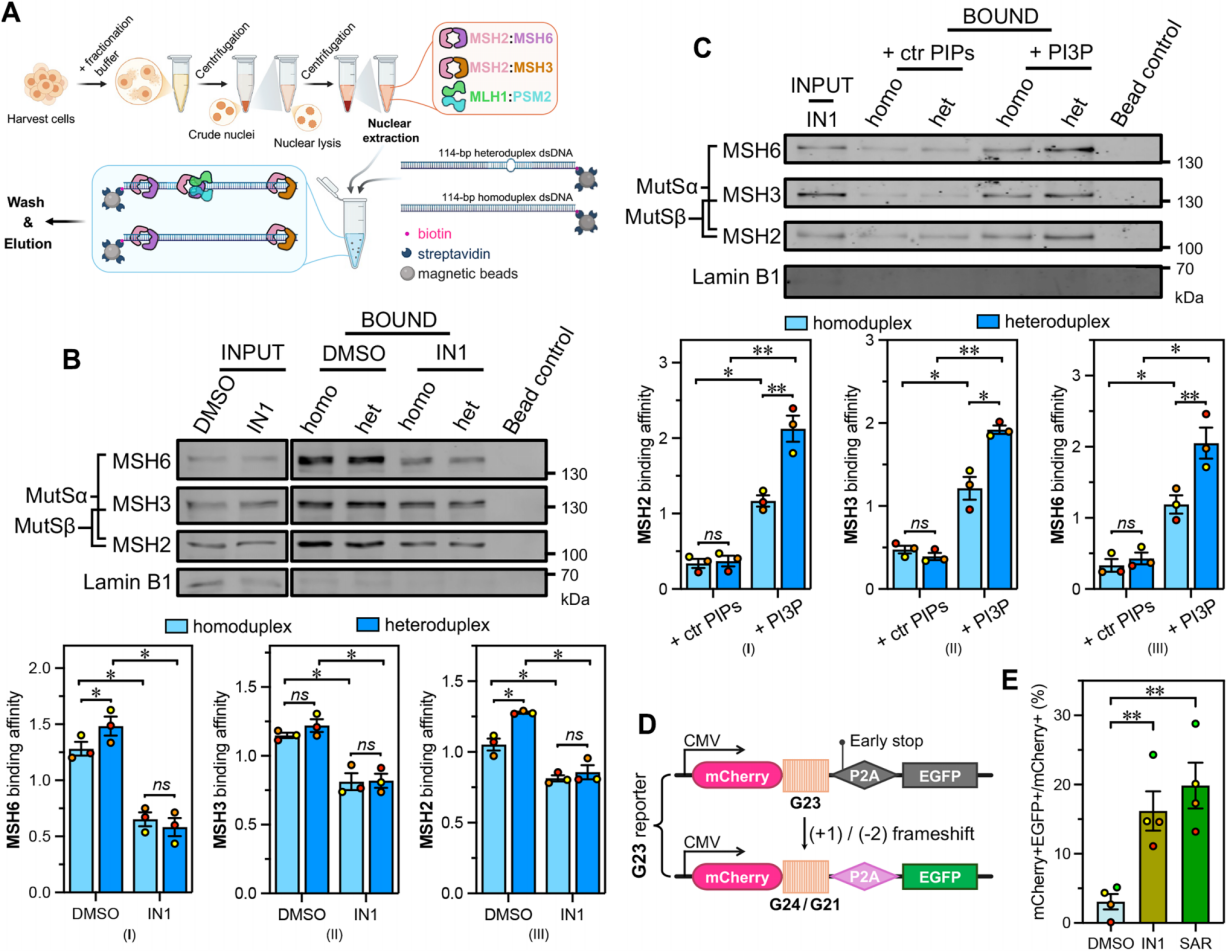
PI3P promotes DNA binding of MMR components and supports MMR function. (**A**) Schematic of the *pseudo-in vitro* DNA binding assay. Nuclear extracts were incubated with streptavidin beads coupled to 114-bp biotinylated dsDNA containing either a G:T mismatch (heteroduplex) or a G:C match (homoduplex). Empty beads (not coupled to dsDNA) served as non-specific binding controls. (**B**) Immunoblot analysis of *pseudo-in vitro* DNA binding assays using nuclear extracts from DMSO- or IN1-treated HeLa cells. Quantification of relative DNA-binding affinity (bead-bound protein normalized to input) for MSH6 (I), MSH3 (II), and MSH2 (III) is shown below. Light blue: homoduplex; dark blue: heteroduplex. *n* = 3 biological replicates. *P < .05. (**C**) Immunoblot analysis of *pseudo-in vitro* DNA binding assays using PI3P-deficient nuclear extracts from IN1-treated cells supplemented with control phosphoinositides (ctr PIPs) or PI3P (10 μM). Quantification of relative DNA-binding affinity (bead-bound protein normalized to input) for MSH6 (I), MSH3 (II), and MSH2 (III) is shown below. Light blue: homoduplex; dark blue: heteroduplex. *n* = 3 biological replicates. *P < .05, **P < .01. (**D**) Schematic of the dual-fluorescence MSI reporter system. The G23 MSI reporter contains a 23-guanine mononucleotide tract between mCherry and out-of-frame EGFP; instability within the 23-guanine monotract can restore EGFP expression. The N23 control reporter replaces the 23-guanine mononucleotide tract with a non-repetitive 23-nucleotide sequence as shown in Supplementary Fig. S3A. (**E**) Flow cytometric quantification of MSI status in G23 MSI reporter-expressing HeLa cells treated with DMSO, IN1 (1 μM), or SAR405 (2 μM) for 32 h. MSI status is calculated as the mCherry^+^EGFP^+^/mCherry^+^ ratio. Corresponding representative dot plots are provided in Supplementary Fig. S3E. *n* = 4 biological replicates. **P < .01.

With impaired MMR recognition complex assembly in the absence of PI3P, we wondered whether PI3P deficiency compromised DNA association of the MMR machinery. To test this, we employed a *pseudo-in vitro* DNA binding assay adapted from established methods [56, 57]. Briefly, nuclear extracts were incubated with streptavidin beads coupled to 114-bp biotinylated dsDNA containing either a G:T mismatch (heteroduplex) or a G:C match (homoduplex), allowing recovery of DNA-associated nuclear proteins, including MMR components, through isolation of the streptavidin beads (Fig. 3A). Nuclear extracts from PI3P-containing DMSO-treated cells bound both substrates, with MutSα components MSH2 and MSH6 showing modest preference for the heteroduplex, consistent with mismatch recognition preference of MutSα [57–59]. Although this degree of substrate preference was less pronounced than those reported in purified reconstituted systems [60–62], this likely reflects the nature of the nuclear extract-based *pseudo-in vitro* assay used here, in which homoduplex and heteroduplex substrates are assessed separately and MMR complexes are examined outside a fully defined biochemical system [63–65]. In contrast, PI3P-deficient nuclear extracts from IN1-treated cells showed reduced binding of MSH2 and MSH6 to both substrates, together with a reduction in the modest preference for the heteroduplex (Fig. 3B). Similar effects were observed in PI3P-deficient nuclear extracts from Vps34-depleted cells (Supplementary Fig. S2D). Moreover, supplementation of PI3P-deficient nuclear extracts with exogenous PI3P, compared with control phosphoinositides, increased association of MSH2, MSH6, and MSH3 with both DNA substrates in nuclear extracts prepared from IN1-treated cells (Fig. 3C). Similar effects were observed when PI3P was added to PI3P-deficient nuclear extracts from Vps34-depleted cells (Supplementary Fig. S2E). Together, these data demonstrate that PI3P can promote association of MMR recognition complex, especially MutSα heterodimer, with substrate DNA.

Functional MMR is essential for maintaining microsatellite stability. To assess whether PI3P depletion impairs MMR function, we adapted a dual-fluorescence MSI reporter based on a previous system [66]. The G23 MSI reporter (mCherry-G23-P2A-EGFP) places a 23-guanine mononucleotide tract and a self-cleaving P2A peptide between an upstream mCherry and a downstream out-of-frame EGFP (Fig. 3D), such that EGFP expression requires length change within the 23-guanine mononucleotide repeat arising from unrepaired replication slippage due to dysfunctional MMR [67]. Thus, the mCherry^+^EGFP^+^/mCherry^+^ ratio provides a quantitative readout of mononucleotide MSI and, by extension, is more directly informative for MMR functions typically associated with MutSα. The N23 control reporter replaces the G23 tract with a non-repetitive 23-nt sequence whose replication fidelity does not primarily depend on MMR (Supplementary Fig. S3A). As expected, MSH2 depletion specifically increased the mCherry^+^EGFP^+^ population in G23 MSI reporter-expressing cells, confirming MMR dependence of the readout (Supplementary Fig. S3B–D). PI3P depletion through either pharmacological or genetic perturbation of Vps34 increased the fraction of mCherry^+^EGFP^+^ population in cells carrying the G23 MSI reporter without affecting those carrying the N23 control reporter (Fig. 3E and Supplementary Fig. S3E and F), consistent with impaired MMR function. Together, these results show that nuclear PI3P promotes MMR recognition complex assembly and DNA association, while depletion of nuclear PI3P compromises MMR function and increases MSI at mononucleotide repeats, supporting a role for nuclear PI3P as a novel lipid regulator of MMR.

### The nuclear Beclin-1/Vps34 complex generates PI3P to regulate MMR independent of autophagy

**Figure 4.**
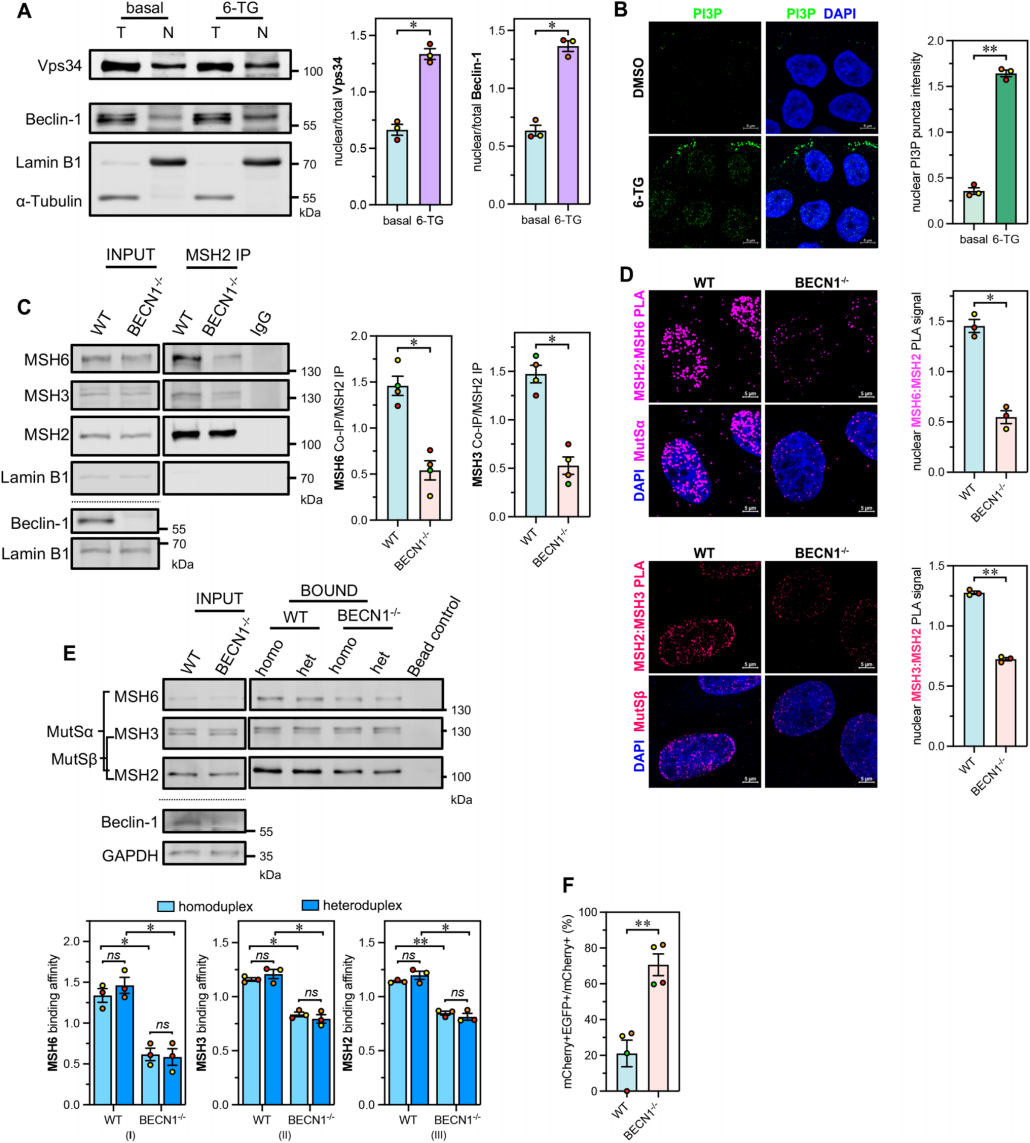
Vps34 modulator Beclin-1 participates in MMR regulation. (**A**) Immunoblot analysis of Beclin-1 and Vps34 levels in nuclear fractions from HeLa cells 24 h after 30 μM 6-TG exposure for 3 h. Quantifications of nuclear/total Vps34 and Beclin-1 are shown on the right. *n* = 3 biological replicates. *P < .05. (**B**) Immunofluorescence analysis of nuclear PI3P puncta in HeLa cells 24 h after 30 μM 6-TG exposure for 3 h, using DMSO as vehicle control. PI3P is probed with anti-PI3P antibody. Quantification of nuclear PI3P puncta intensity is shown on the right. *n* = 3 biological replicates. **P < .01. (**C**) Immunoblot analysis of MSH2 IP from nuclear extracts of WT and BECN1 knockout (BECN1^−/−^) HeLa cells. Quantification of MSH6/MSH2 and MSH3/MSH2 ratios is shown on the right. *n* = 4 biological replicates. *P < .05. (**D**) PLA detecting MutSα (top) and MutSβ (bottom) in WT and BECN1^−/−^ HeLa cells. Quantification of nuclear PLA foci per cell is shown on the right. Scale bar: 5 μm. *n* = 3 biological replicates. *P < .05, **P < .01. (**E**) Immunoblot analysis of *pseudo-in vitro* DNA binding assays using nuclear extracts from WT and BECN1^−/−^ HeLa cells. Quantification of relative DNA-binding affinity for MSH6 (I), MSH3 (II), and MSH2 (III) is shown below. Light blue: homoduplex; dark blue: heteroduplex. *n* = 3 biological replicates. *P < .05, **P < .01. (**F**) Flow cytometric quantification of MSI status in G23 MSI reporter-expressing WT and BECN1^−/−^ cells. MSI status is calculated as the mCherry^+^EGFP^+^/mCherry^+^ ratio. *n* = 4 biological replicates. **P < .01.

Because PI3P promotes MMR recognition complex assembly and DNA association, we next investigated how this nuclear PI3P pool is generated. Beclin-1 and Vps34 are canonical components of the class III PI3-kinase complex that produces PI3P on cytoplasmic membranous structures to regulate autophagy [23, 68–70], while recent studies have suggested that Beclin-1 can also localize to the nucleus and safeguard genome integrity [33–35, 54]. Exposure to 6-TG, a thiopurine whose cytotoxicity depends on functional MMR, substantially increased the nuclear levels of Beclin-1 and Vps34 and was accompanied by a corresponding increase in the nuclear PI3P signal (Fig. 4A and B), supporting the idea that genotoxic stress promotes formation of a nuclear Beclin-1/Vps34/PI3P axis under MMR-engaging conditions.

To test whether Beclin-1 modulates MMR, we used *BECN1* knockout (BECN1^−/−^) HeLa cells and their WT control (Supplementary Fig. S4A). As with PI3P depletion, loss of Beclin-1 did not alter nuclear abundance of MSH2, MSH3, or MSH6 (Supplementary Fig. S4A), but impaired MutSα and MutSβ assembly, as revealed by markedly reduced immunoprecipitations of MSH6 and MSH3 by MSH2 in nuclear extracts from BECN1^−/−^ cells compared with WT (Fig. 4C). Nuclear MutSα and MutSβ heterodimers were markedly reduced when detected *in situ* by PLA in BECN1^−/−^ cells (Fig. 4D and Supplementary Fig. S4B and C). Nuclear extracts from BECN1^−/−^ cells also displayed reduced binding of MSH2, MSH6, and MSH3 to both homoduplex and heteroduplex substrates in the *pseudo-in vitro* DNA binding assay (Fig. 4E), similar to PI3P-deficient extracts. Accordingly, BECN1^−/−^ cells exhibited elevated MSI as measured by the G23 MSI reporter (Fig. 4F and Supplementary Fig. S4D), confirming that Beclin-1 deficiency impairs MMR function.

**Figure 5.**
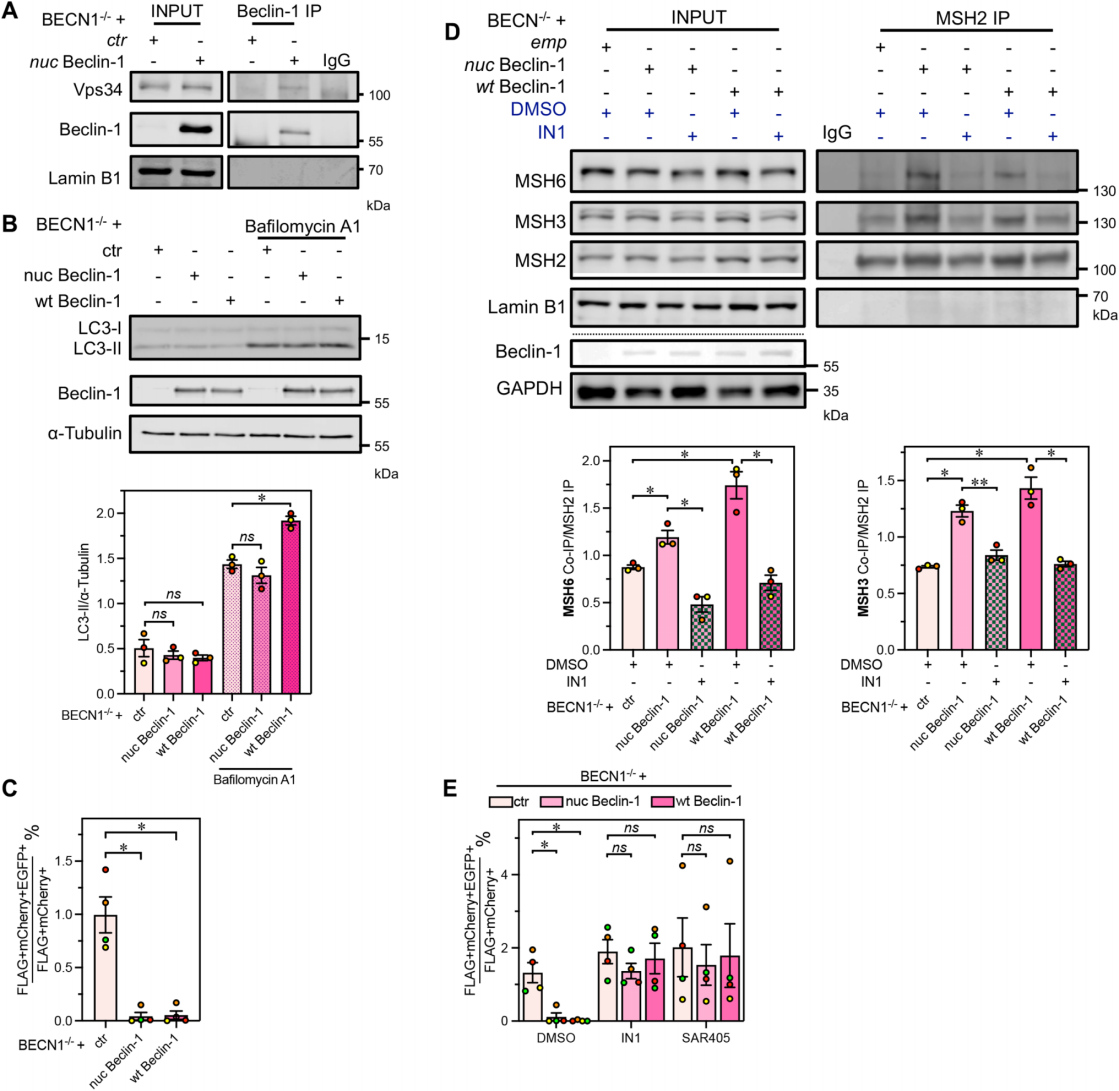
The nuclear Beclin-1/Vps34 complex generates PI3P to regulate MMR independent of autophagy. (**A**) Immunoblot analysis of Beclin-1 IP from nuclear extracts of BECN1^−/−^ cells reconstituted with control vector (ctr) or nuclear Beclin-1 (nuc Beclin-1), demonstrating Vps34 co-immunoprecipitation with Beclin-1. Representative of *n* = 3 independent experiments. (**B**) Immunoblot analysis of LC3-II levels in BECN1^−/−^ cells reconstituted with control vector, nuc Beclin-1, or WT Beclin-1 (wt Beclin-1) under basal conditions or following bafilomycin A1 treatment (400 nM, 4 h). Quantifications of LC3-II/α-tubulin are shown on the right. *n* = 3 biological replicates. *P < .05. (**C**) Flow cytometric quantification of MSI status in G23 MSI reporter-expressing BECN1^−/−^ cells reconstituted with control vector, nuclear Beclin-1, or WT Beclin-1. Transfected cells were gated by the FLAG tag. MSI status is calculated as the FLAG^+^mCherry^+^EGFP^+^/FLAG^+^mCherry^+^ ratio. Corresponding representative dot plots are provided in Supplementary Fig. S5C. *n* = 4 biological replicates. *P < .05. (**D**) Immunoblot analysis of MSH2 IP from nuclear extracts of BECN1^−/−^ cells reconstituted with control vector, nuclear Beclin-1, or WT Beclin-1, followed by treatment with DMSO or IN1 (1 μM, 16 h). Quantification of MSH6/MSH2 and MSH3/MSH2 ratios is shown below. *n* = 3 biological replicates. *P < .05, **P < .01. (**E**) Flow cytometric quantification of MSI status in G23 MSI reporter-expressing BECN1^−/−^ cells reconstituted with control vector, nuclear Beclin-1, or WT Beclin-1, followed by treatment with DMSO, IN1 (1 μM), or SAR405 (2 μM) for 32 h. Transfected cells were gated by the FLAG tag. MSI status is calculated as the FLAG^+^mCherry^+^EGFP^+^/FLAG^+^mCherry^+^ ratio. Corresponding representative dot plots are provided in Supplementary Fig. S5C. *n* = 4 biological replicates. *P < .05.

To discriminate the nuclear role of Beclin-1 from its cytoplasmic, autophagy-related functions, we engineered a nuclear Beclin-1 construct combining L184A/L187A mutations in its nuclear export sequence with a C-terminal nuclear localization sequence (hereinafter referred to as nuclear Beclin-1), along with a WT Beclin-1 construct and an empty control vector. A C-terminal FLAG tag enabled immunofluorescent detection of subcellular localization (Supplementary Fig. S4E). Beclin-1 forms complexes with Vps34 in the nucleus, as evident from the recovery of Vps34 by Beclin-1 in nuclear extracts from BECN1^−/−^ cells overexpressing nuclear Beclin-1 (Fig. 5A). Meanwhile, MMR proteins were found enriched in nuclear Beclin-1 interactome (Supplementary Fig. S4F and Supplementary Table S1). Moreover, unlike WT Beclin-1, nuclear Beclin-1 did not restore autophagic flux in BECN1^−/−^ cells as measured by LC3-II level under basal and bafilomycin A1-treated conditions, supporting an autophagy-independent function of the nuclear Beclin-1/Vps34 complex (Fig. 5B). Reconstitution of BECN1^−/−^ cells with nuclear Beclin-1 restored MutSα and MutSβ assembly to a similar extent as WT Beclin-1, as assessed by MSH2 immunoprecipitation, without affecting the nuclear abundance of MSH2, MSH3, or MSH6 (Supplementary Fig. S5A). Moreover, both WT and nuclear Beclin-1 promoted DNA substrate association of MMR components in BECN1^−/−^ cells (Supplementary Fig. S5B). Importantly, both WT and nuclear Beclin-1 also reduced MSI at mononucleotide repeats in BECN1^−/−^ cells to a comparable extent, indicating functional complementation of the MMR defect (Fig. 5C and Supplementary Fig. S5C). However, upon Vps34 inhibition with IN1 or SAR405, the rescue in MutS complex assembly and MSI was abolished (Fig. 5D and E, Supplementary Fig. S5C), indicating that the functional effect of nuclear Beclin-1 depends on Vps34 kinase activity and PI3P production. Together, these data support the view that Beclin-1 acts with Vps34 to generate PI3P in the nucleus and thereby promote MMR-associated processes. These findings identify a previously unrecognized, autophagy-independent nuclear role for the Beclin-1/Vps34 complex in genome maintenance, through PI3P-mediated regulation of MMR.

### Beclin-1/Vps34 deficiency impairs MMR-dependent genome maintenance and confers resistance to genotoxic stress

**Figure 6.**
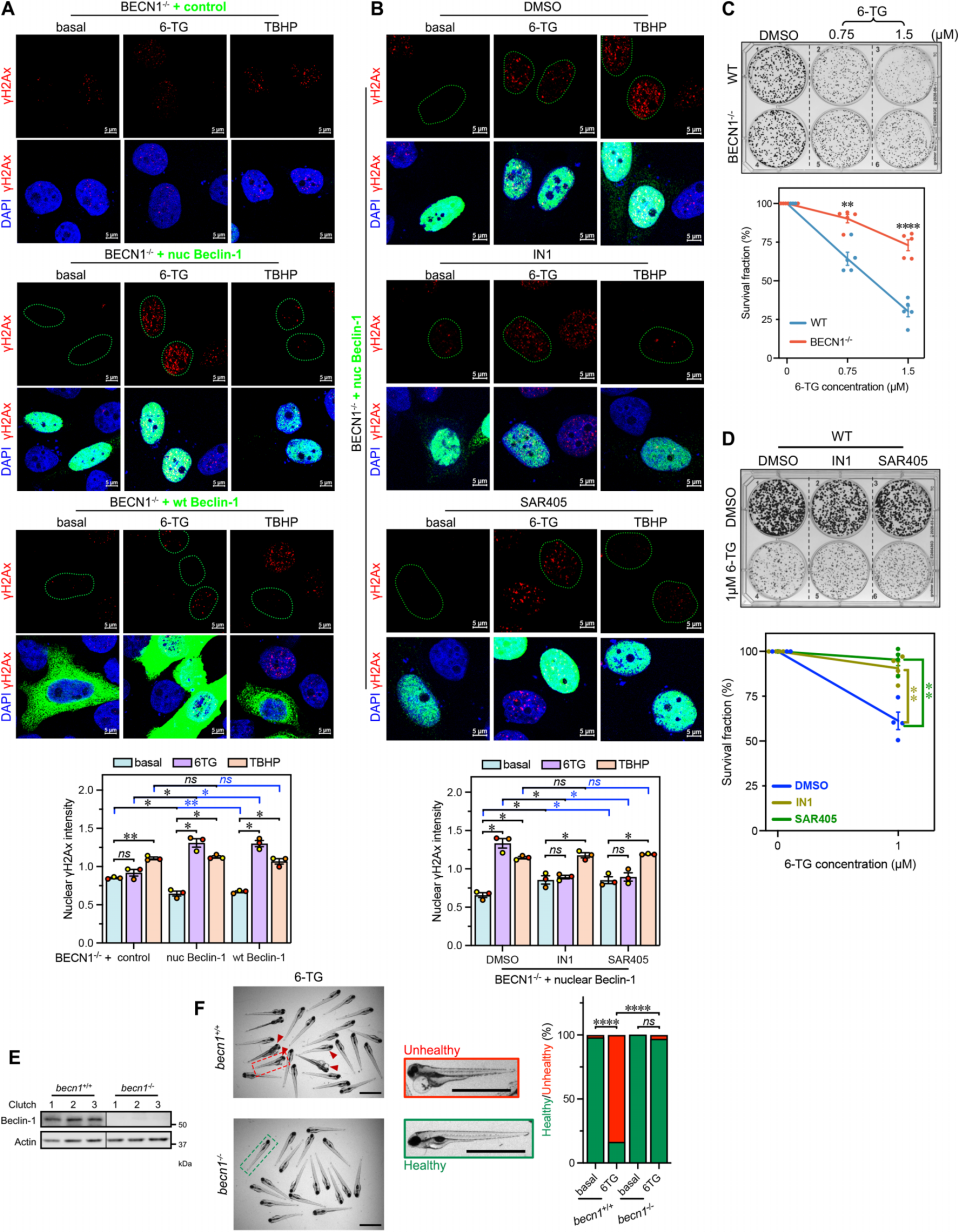
Beclin-1/Vps34 deficiency impairs MMR-dependent DNA damage signaling and confers resistance to genotoxic stress. (**A**) Immunofluorescence analysis of γH2AX foci in BECN1^−/−^ cells reconstituted with control vector, nuclear Beclin-1, or WT Beclin-1, under basal conditions or following treatment with 6-TG or tert-butyl hydroperoxide (TBHP, 100 μM, 45 min). Scale bar: 5 μm. Quantification of γH2AX foci per nucleus is shown at the bottom. *n* = 3 biological replicates. *P < .05, **P < .01. (**B**) Immunofluorescence analysis of γH2AX foci in BECN1^−/−^ cells reconstituted with nuclear Beclin-1 and treated with DMSO, IN1, or SAR405, under basal conditions or following treatment with 6-TG or TBHP. Scale bar: 5 μm. Quantification of γH2AX foci per nucleus is shown at the bottom. *n* = 3 biological replicates. *P < .05. (**C**) Clonogenic survival assay of WT and BECN1^−/−^ HeLa cells under chronic 6-TG exposure (0.75 μM or 1.5 μM, 14 days). Representative colony images (top) and quantification of surviving colony fraction (bottom) are shown. *n* = 5 biological replicates. **P < .01, ****P < .0001. (**D**) Clonogenic survival assay of HeLa cells co-treated with 6-TG (1 μM) and DMSO, IN1 (1 μM for 1 day and maintain 100 nM), or SAR405 (2 μM for 1 day and maintain 200 nM) for 14 days. Representative colony images (top) and quantification (bottom) are shown. *n* = 4 biological replicates. **P < .01. (**E**) Immunoblot confirming loss of Beclin-1 protein in homozygous *becn1* knockout zebrafish (*becn1^−/−^*) compared with WT control fish (*becn1^+/+^*). (**F**) Toxicity assay to evaluate the genotoxic effect of 600 μM 6-TG in *becn1^+/+^* and *becn1^−/−^* zebrafish. Representative images of clutches are shown on the left. Red arrowheads indicate morphologically abnormal fish. Data are represented as the percentage of healthy versus unhealthy zebrafish scored at 5 d.p.f. *n* = 3 independent clutches per group. Statistical significance was assessed using Chi-square test. ****P < .0001. Scale bar is 2 mm for all images.

Having established that nuclear PI3P generated by the Beclin-1/Vps34 complex can promote MutSα and MutSβ assembly and mismatch engagement, we next examined how disruption of this pathway affects MMR-dependent DNA damage signaling and genotoxic responses. The thiopurine 6-TG incorporates into DNA and, when present in the template strand, triggers futile MMR cycles that generate strand breaks and ultimately induce apoptosis [11, 71]. Following exposure to 6-TG, γH2AX foci accumulated in MMR-proficient cells, whereas such γH2AX induction was diminished when MMR was dampened upon MSH2 depletion, consistent with previous reports [72–75]. γH2AX formation in response to tert-butyl hydroperoxide (TBHP) remained unaffected by MMR status, indicating unchanged vulnerability to DNA damage, further confirming that γH2AX induction following 6-TG exposure depends on functional MMR (Supplementary Fig. S6A). Beclin-1 deficiency increased genome instability at basal levels, aligning with its reported role in safeguarding genome stability [34, 36, 76]. However, BECN1^−/−^ cells displayed blunted γH2AX induction in response to 6-TG compared with WT cells (Supplementary Fig. S6B). Comparable impairment was observed upon pharmacological inhibition of Vps34 with IN1 or SAR405 (Supplementary Fig. S6C) or siRNA-mediated Vps34 depletion (Supplementary Fig. S6D). Nuclear and WT Beclin-1 robustly restored γH2AX induction in BECN1^−/−^ cells following 6-TG exposure, whereas the control vector failed to do so (Fig. 6A). Importantly, γH2AX induction in response to 6-TG in nuclear Beclin-1-reconstituted BECN1^−/−^ cells was abolished in the absence of PI3P upon IN1 or SAR405 treatment (Fig. 6B). The complete loss of nuclear Beclin-1-mediated rescue upon Vps34 inhibition indicates that the enhanced γH2AX response to 6-TG by nuclear Beclin-1 depends on Vps34 activity and PI3P production rather than on nuclear Beclin-1 expression alone. These findings demonstrate that the nuclear Beclin-1/Vps34 is the upstream generator of PI3P, which regulates MMR and further contributes to MMR-dependent DNA damage signaling.

Because defective MMR-triggered signaling reduces thiopurine sensitivity, we next evaluated the impact of Beclin-1/Vps34 loss on 6-TG cytotoxicity. In clonogenic survival assays, BECN1^−/−^ cells showed markedly improved survival under chronic 6-TG exposure compared with WT cells (Fig. 6C). Similarly, Vps34 inhibition with both IN1 and SAR405 increased survival under chronic 6-TG exposure (Fig. 6D), closely mirroring the phenotype of MMR-deficient cells. To further determine whether this regulatory axis operates *in vivo*, we used zebrafish as a tractable vertebrate model that enables organismal-level assessment of genotoxic stress responses and associated morphological outcomes. We generated zebrafish lines with homozygous *becn1* knockout (*becn1^−/−^*) and WT siblings (*becn1^+/−^*) (Fig. 6E), and zebrafish transiently depleted for Beclin-1 via *becn1*-targeting CRISPR guides (Supplementary Fig. S7A). In the absence of Beclin-1, zebrafish exhibited improved survival and healthier overall morphology under chronic 6-TG exposure, as evidenced by fewer fish with pericardial edemas and reduced body size (Fig. 6F and Supplementary Fig. S7B).

Together, these results establish that loss of Beclin-1/Vps34-mediated nuclear PI3P synthesis disrupts MMR-dependent genome maintenance and confers resistance to thiopurine-induced cytotoxic stress in both cultured cells and whole organisms.

## Discussion

This study identifies nuclear PI3P as a lipid regulator of DNA MMR and reveals an autophagy-independent nuclear function for the Beclin-1/Vps34 complex. We show that Vps34 and Beclin-1 relocalize to the nucleus in response to MMR-engaging lesions, where they generate a nuclear PI3P pool that is closely associated with MutSα (MSH2:MSH6) and MutSβ (MSH2:MSH3) MMR recognition complexes. Our results support a model where nuclear PI3P regulates MMR through promoting MMR recognition complex assembly and their association with substrate DNA, further contributing to MMR functional integrity and microsatellite stability, while also indirectly enabling MMR-dependent DNA damage signaling that could govern cell fate. Disruption of the Beclin-1/Vps34/PI3P axis was shown to confer survival advantage to 6-TG-induced cytotoxicity in cells and *in vivo*. Together, these findings broaden the functional repertoire of the Beclin-1/Vps34 complex beyond its canonical roles in autophagy and endosomal trafficking and support a role for nuclear phosphoinositide signaling in genome maintenance.

MMR has traditionally been viewed as a protein-centric pathway, controlled by transcriptional regulation, post-translational modifications, protein–protein and protein-chromatin interactions, and cell-cycle cues [12, 77, 78]. The identification of PI3P as a regulator for MMR recognition complex assembly and substrate DNA association introduces a lipid dimension to this regulatory framework. PI3P does not alter the nuclear abundance of MSH2, MSH3, or MSH6, but instead acts at the level of recognition complex formation and substrate engagement. The ability of exogenous PI3P to promote MutS assembly and DNA engagement in a cell-free system supports a biochemical requirement for this lipid in constructing a functional MMR recognition module. A parsimonious model is that PI3P defines a permissive nuclear microenvironment for the MMR recognition complexes, either by stabilizing a conformation competent for DNA loading or by recruiting scaffolding factors that bridge PI3P to the MMR machinery. Although MutSα and MutSβ lack canonical FYVE, PX, or PROPPIN domains [27, 44, 46], basic or amphipathic surfaces within MutS components or associated proteins may function as non-canonical PI3P-interacting interfaces. Defining such interfaces, and determining whether MutS complexes bind PI3P directly or through adapter proteins, will be an important direction for future structural and biochemical work. In particular, the PI3P pulldown and nuclear-extract reconstitution data are consistent with either direct lipid engagement by MutS complexes via non-canonical PI3P-binding motifs, or PI3P-dependent recruitment/organization of auxiliary factors that promote MutSα and MutSβ heterodimerization and/or DNA loading; distinguishing these models will require mapping PI3P-responsive regions in the relevant MMR proteins and testing purified components in defined systems.

These findings also extend the emerging view that nuclear phosphoinositides contribute to genome regulation. Phosphoinositides such as PI(4,5)P_2_ and PI(3,4,5)P_3_ have been implicated in chromatin remodeling, transcriptional control, and certain DNA damage responses [29–32, 79], whereas PI3P has been predominantly studied on early endosomes and autophagic membranes. Showing that PI3P forms discrete nuclear puncta, resides in close proximity to MutSα and MutSβ, and promotes MMR initiation suggests that nuclear phosphoinositide pools encode a “lipid layer” of information that shapes genome surveillance. In addition to their canonical post-replicative repair function, MutS complexes can also participate in non-canonical contexts, including immunoglobulin somatic hypermutation and class-switch recombination [80, 81]. Our finding that nuclear PI3P modulates MutS complex-associated processes therefore raises the possibility that PI3P-dependent regulation could influence MMR outputs beyond classical replication-error correction. Meanwhile, recent studies have demonstrated interactions between PI3P and other repair factors, including flap endonuclease-1 (FEN1) and RAD51 [49], supporting the possibility that nuclear PI3P coordinates multiple repair pathways beyond MMR. Together with broader evidence linking nuclear Beclin-1 to DNA damage responses and double-strand break repair [34], this raises the possibility that nuclear PI3P may influence additional repair pathways beyond MMR, although the pathway scope and mechanism of such regulation remain to be defined.

In addition, a recent report has suggested a nuclear Vps34-containing complex that constrains γH2A spreading around double-strand breaks, positioning Vps34 at the interface of chromatin topology and damage signaling (bioRxiv: www.biorxiv.org/content/10.1101/2022.03.09.482075v1). Together with our findings, these observations support a potential theme in which nuclear Vps34 complexes, via PI3P production, could contribute to the spatial and functional organization of DNA repair pathways, despite differences in lesion types, protein partners, and downstream outcomes.

In summary, this work demonstrates that a nuclear Beclin-1/Vps34 complex generates PI3P to regulate DNA MMR by promoting MutSα and MutSβ MMR recognition complex assembly and DNA association. This lipid-mediated mechanism safeguards microsatellite stability, shapes cellular responses to genotoxic stress, and broadens the conceptual scope of both nuclear phosphoinositide signaling and Beclin-1/Vps34 biology.

## Supplementary Material

gkag696_Supplemental_Files

## Data Availability

All data reported in this paper will be shared by the corresponding contact, David C. Rubinsztein (dcr1000@cam.ac.uk) upon request. The mass spectrometry proteomics data have been deposited to the ProteomeXchange Consortium via the PRIDE partner repository with the dataset identifiers. PRIDE: PXD066473.
