# Supplementary material for: Nuclear PI3P produced by the Beclin-1/Vps34 complex regulates DNA mismatch repair
Source: Nucleic Acids Res. 2026 Jul 14;54(13):gkag696. doi: 10.1093/nar/gkag696 (PMC13366062; doi:10.1093/nar/gkag696)
Supplement: gkag696_Supplemental_Files [file gkag696_supplemental_files.zip › SUPP_260512_NAR_Clear-fixed.pdf]

Supplementary data for:

Nuclear PI3P produced by the Beclin-1/Vps34 complex regulates DNA mismatch repair

Xinyi Li[1,2], Mariella Vicinanza[1], Ana Lopez[1,2,3], Beatrice Paola Festa[1,2], Lars Schlotawa[1,3], Antonio Daniel Barbosa[1,2], Michael Takla[1,2], Gabriel Balmus[4,5], Angeleen Fleming[1,2,3], and David C. Rubinsztein[1,2,*]

[1] Cambridge Institute for Medical Research (CIMR), Department of Genomic Medicine, University of Cambridge, Cambridge CB2 0XY, UK

[2] UK Dementia Research Institute, Cambridge Institute for Medical Research (CIMR), University of Cambridge, Cambridge CB2 0XY, UK

[3] Department of Physiology, Development and Neuroscience, University of Cambridge, Cambridge, UK

[4] UK Dementia Research Institute at University of Cambridge, Department of Clinical Neurosciences, University of Cambridge, Cambridge, UK

[5] Department of Molecular Neuroscience, Transylvanian Institute of Neuroscience, Cluj-Napoca, Romania

* To whom correspondence should be addressed. Email: dcr1000@cam.ac.uk

This document includes:

Supplementary Figures 1 to 7

Supplementary Table Legend

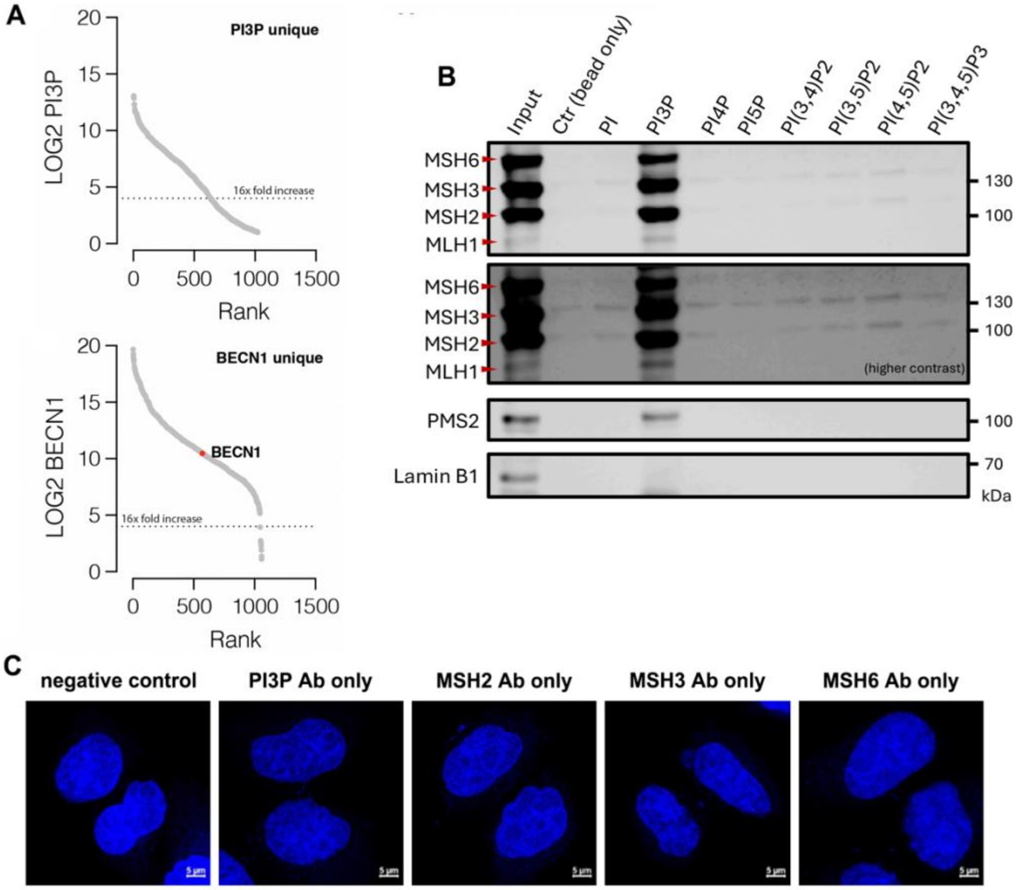

Supplementary Fig. 1: Nuclear PI3P associates with MMR

(A) Rank plot showing proteins uniquely enriched in PI3P and Beclin1 nuclear pulldown proteomics (16X enrichment threshold shown).

(B) Immunoblot analysis of pulldowns in nuclear extracts using a panel of phosphoinositide-conjugated beads, including Ctr (bead only), PI-, PI3P-, PI4P-, PI5P-, PI(3,4)P2-, PI(4,5)P2-, PI(3,5)P2- and PI(3,4,5)P3-conjugated beads. MMR proteins (MSH2, MSH3, MSH6, PMS2 and MLH1) exhibited highest affinity for PI3P-conjugated beads, whereas only MSH3 and MSH2 weakly interact with other phosphoinositide species. Representative of n = 4 independent experiments.

(C) Antibody specificity controls for proximity ligation assay (PLA) in HeLa cells performed by omitting all primary antibodies (negative control) or by including only single primary antibodies against PI3P, MSH2, MSH6, or MSH3. No PLA signal was detected under any condition, confirming that the PLA signals observed throughout the study arise specifically from spatial proximity between paired target molecules. Scale bar: 5 μm.

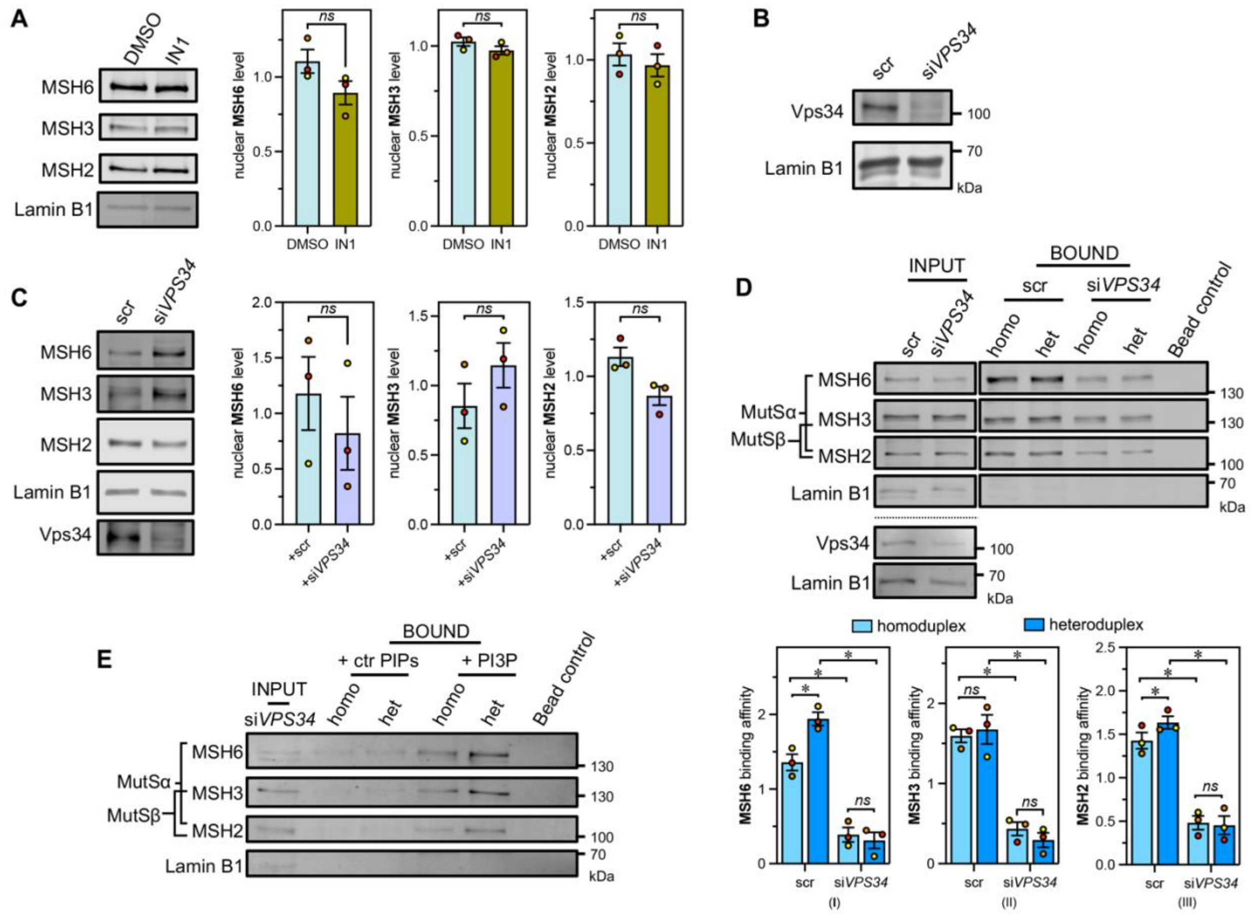

Supplementary Fig. 2: PI3P supports MMR recognition complex assembly and DNA engagement

(A) Representative immunoblot and quantification of MSH2, MSH3, and MSH6 nuclear abundance in HeLa cells treated with DMSO or IN1 (1 µM, 16 h), as shown in the nuclear input in Fig. 2A. Quantification is shown on the right. n = 3 biological replicates.

(B) Immunoblot confirming *VPS34* knockdown efficiency with si*VPS34* compared with scrambled siRNA (scr) in HeLa cells.

(C) Representative immunoblot and quantification of MSH2, MSH3, and MSH6 nuclear abundance in HeLa cells transfected with scr or si*VPS34*, as shown in the nuclear input in Fig. 2C. Quantification is shown on the right. n = 3 biological replicates.

(D) Immunoblot analysis of *pseudo-in vitro* DNA binding assays using nuclear extracts from scr- or si*VPS34*-transfected cells. Quantification of relative DNA-binding affinity for MSH6 (I), MSH3 (II), and MSH2 (III) is shown below. Light blue: homoduplex; dark blue: heteroduplex. n = 3 biological replicates. *$p < 0.05$.

(E) Immunoblot analysis of *pseudo-in vitro* DNA binding assays using PI3P-deficient nuclear extracts from siVPS34-transfected cells supplemented with control phosphoinositides (ctr PIPs) or PI3P (10 μM). Representative of n = 3 independent experiments.

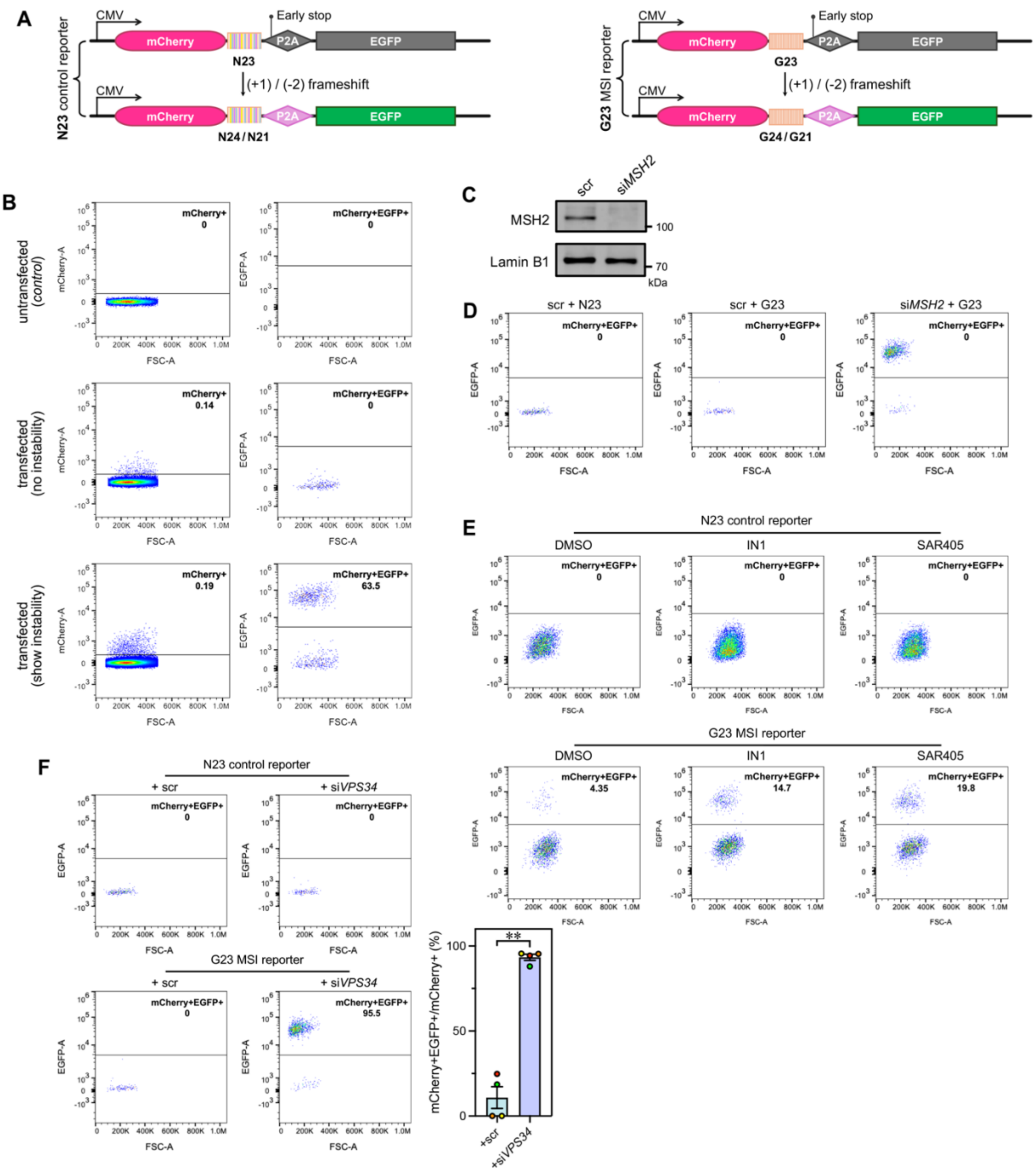

Supplementary Fig. 3: Validation of the dual-fluorescence microsatellite instability reporter system

(A) Schematic of the G23 MSI reporter and N23 control reporter constructs.

(B) Representative flow cytometry plots showing: untransfected cells (top), G23 MSI reporter-expressing cells without microsatellite instability (mCherry^{+} only, middle), and G23 MSI reporter-expressing cells with microsatellite instability (mCherry^{+}EGFP^{+}, bottom).

(C) Immunoblot confirming MSH2 knockdown efficiency with siMSH2 compared with scrambled siRNA (scr) in HeLa cells.

(D) Representative flow cytometry plots of HeLa cells expressing the N23 control reporter or G23 MSI reporter following transfection with scr (MMR-proficient) or siMSH2 (MMR-deficient). MSH2 depletion increases the $mCherry^{+}EGFP^{+}$ population specifically in G23 MSI reporter-expressing cells.

(E) Representative flow cytometry plots of N23 control reporter- or G23 MSI reporter-expressing HeLa cells treated with DMSO, IN1 (1 μM), or SAR405 (2 μM) for 36 h.

(F) Representative flow cytometry plots and quantification of MSI status in HeLa cells treated with scrambled siRNA (scr) or siVPS34. MSI status is calculated as the $mCherry^{+}EGFP^{+}/mCherry^{+}$ ratio. n = 4 biological replicates. $^{**}p < 0.01$.

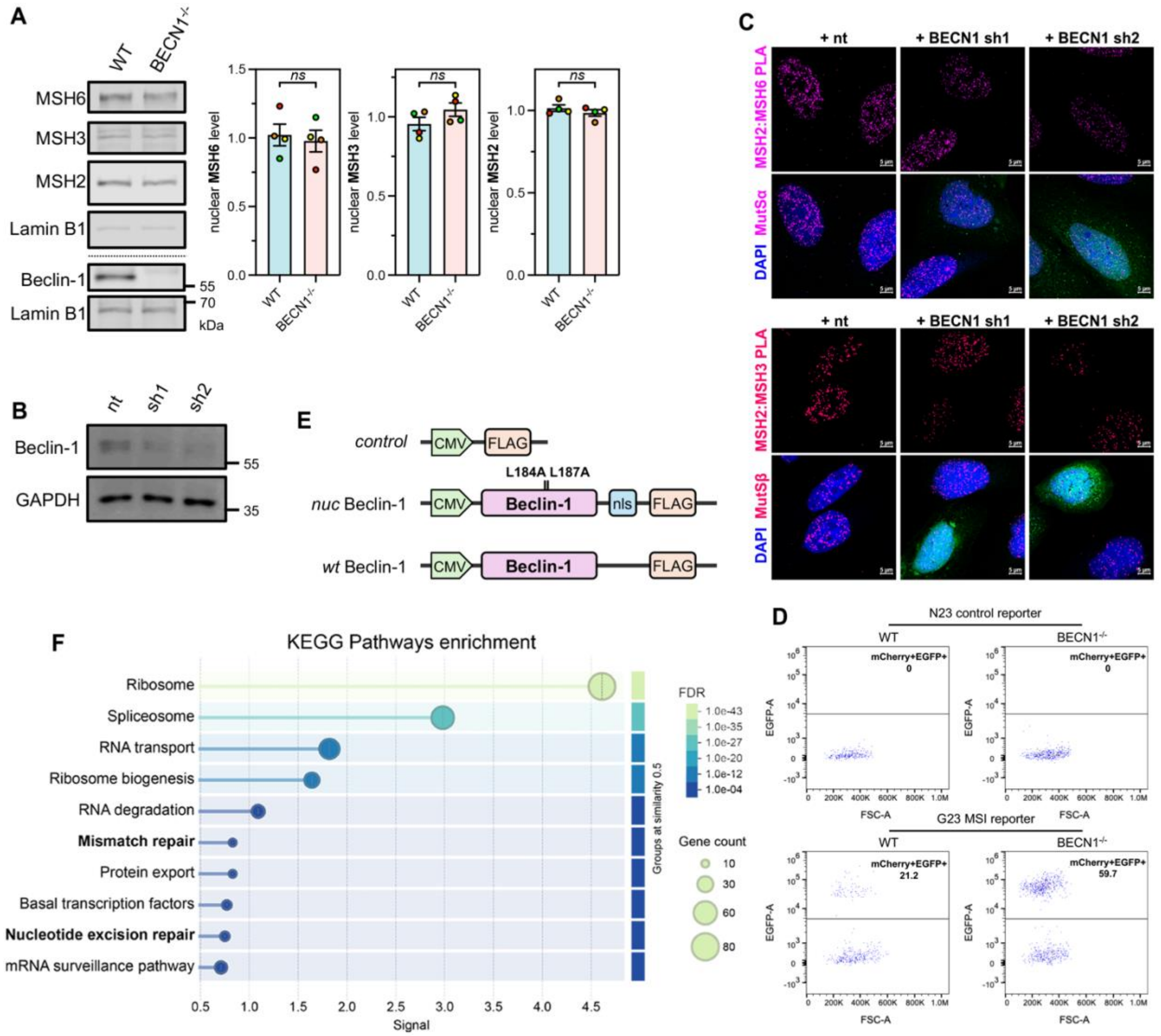

Supplementary Fig. 4: Beclin-1 contributes to MMR regulation

(A) Representative immunoblot and quantification of MSH2, MSH3, and MSH6 nuclear abundance in WT and BECN1$^{-/-}$ cells, as shown in the nuclear input in Fig. 4B. Quantification is shown on the right. n = 3 biological replicates.

(B) Immunoblot confirming knockdown efficiency of two independent *BECN1*-targeting shRNAs (sh1, sh2) compared with non-targeting shRNA (nt) in HeLa cells. GFP signal indicates successful transduction.

(C) PLA detecting MutSα (top) and MutSβ (bottom) in HeLa cells transduced with nt, sh1, or sh2 shRNAs. Scale bar: 5 µm. Representative of n = 3 independent experiments.

(D) Representative flow cytometry plots of N23 control reporter- or G23 MSI reporter-expressing WT and BECN1$^{-/-}$ cells.

(E) Schematic of the control vector, nuclear Beclin-1 (nuc Beclin-1), and wild-type Beclin-1 (wt Beclin-1) expression constructs. Nuclear Beclin-1 combines L184A/L187A mutations with nuclear localization signal to enhance nuclear localization of Beclin-1, as shown in Fig. 4. All constructs bear a C-terminal FLAG epitope.

(F) KEGG pathway enrichment analysis of proteins recovered by Beclin-1 IP from nuclear extracts of $BECN1^{-/-}$ cells expressing nuclear Beclin-1. MMR pathway are highlighted in bold.

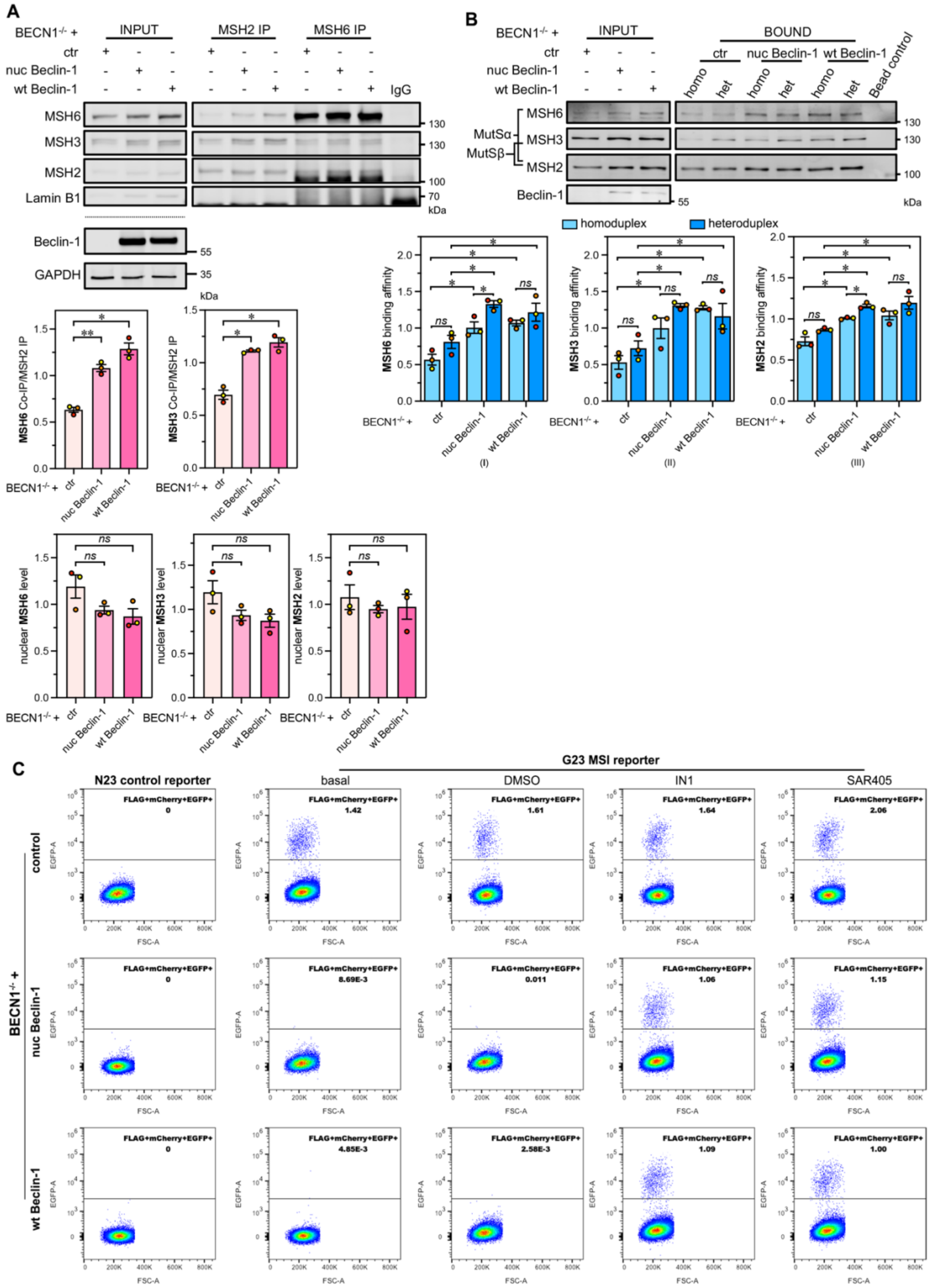
A
BECN1-/- +
INPUT
MSH2 IP
MSH6 IP
ctr
nuc Beclin-1
wt Beclin-1
IgG
MSH6
MSH3
MSH2
Lamin B1
Beclin-1
GAPDH
130
100
70
55
35
kDa
MSH6 Co-IP/MSH2 IP
MSH3 Co-IP/MSH2 IP
nuclear MSH6 level
nuclear MSH3 level
nuclear MSH2 level
ns
B
BOUND
homo
het
Bead control
MutSα
MutSβ
homoduplex
heteroduplex
MSH6 binding affinity
MSH3 binding affinity
MSH2 binding affinity
(I)
(II)
(III)
C
N23 control reporter
G23 MSI reporter
basal
DMSO
IN1
SAR405
control
nuc Beclin-1
wt Beclin-1
FLAG+mCherry+EGFP+
0
1.42
1.61
1.64
2.06
8.69E-3
0.011
1.06
1.15
4.85E-3
2.58E-3
1.09
1.00
EGFP-A
FSC-A

Supplementary Fig. 5: Nuclear Beclin-1 reconstitution restores MMR complex assembly and DNA engagement

(A) Immunoblot analysis of MSH2 IP from nuclear extracts of BECN1$^{-/-}$ cells reconstituted with control vector (ctr), nuclear (nuc) Beclin-1, or wild-type (wt) Beclin-1. Quantification of MSH6/MSH2 and MSH3/MSH2 ratios (upper right) and nuclear MMR protein levels (lower right) are shown. n = 3 biological replicates. *$p < 0.05$, **$p < 0.01$.

(B) Immunoblot analysis of *pseudo-in vitro* DNA binding assays using nuclear extracts from BECN1$^{-/-}$ cells reconstituted with control vector, nuclear or wild-type Beclin-1. Quantification of relative DNA-binding affinity for MSH6 (I), MSH3 (II), and MSH2 (I) is shown below. Light blue: homoduplex; dark blue: heteroduplex. n = 3 biological replicates. *$p < 0.05$.

(C) Representative flow cytometry plots of BECN1$^{-/-}$ cells carrying N23 control reporter or G23 MSI reporter, reconstituted with control vector, nuclear Beclin-1, or wild-type Beclin-1, under basal (unperturbed) condition or followed by treatment with DMSO, IN1 (1 μM), or SAR405 (2 μM) for 32 h. Transfected cells were gated by the FLAG tag. n = 4 biological replicates.

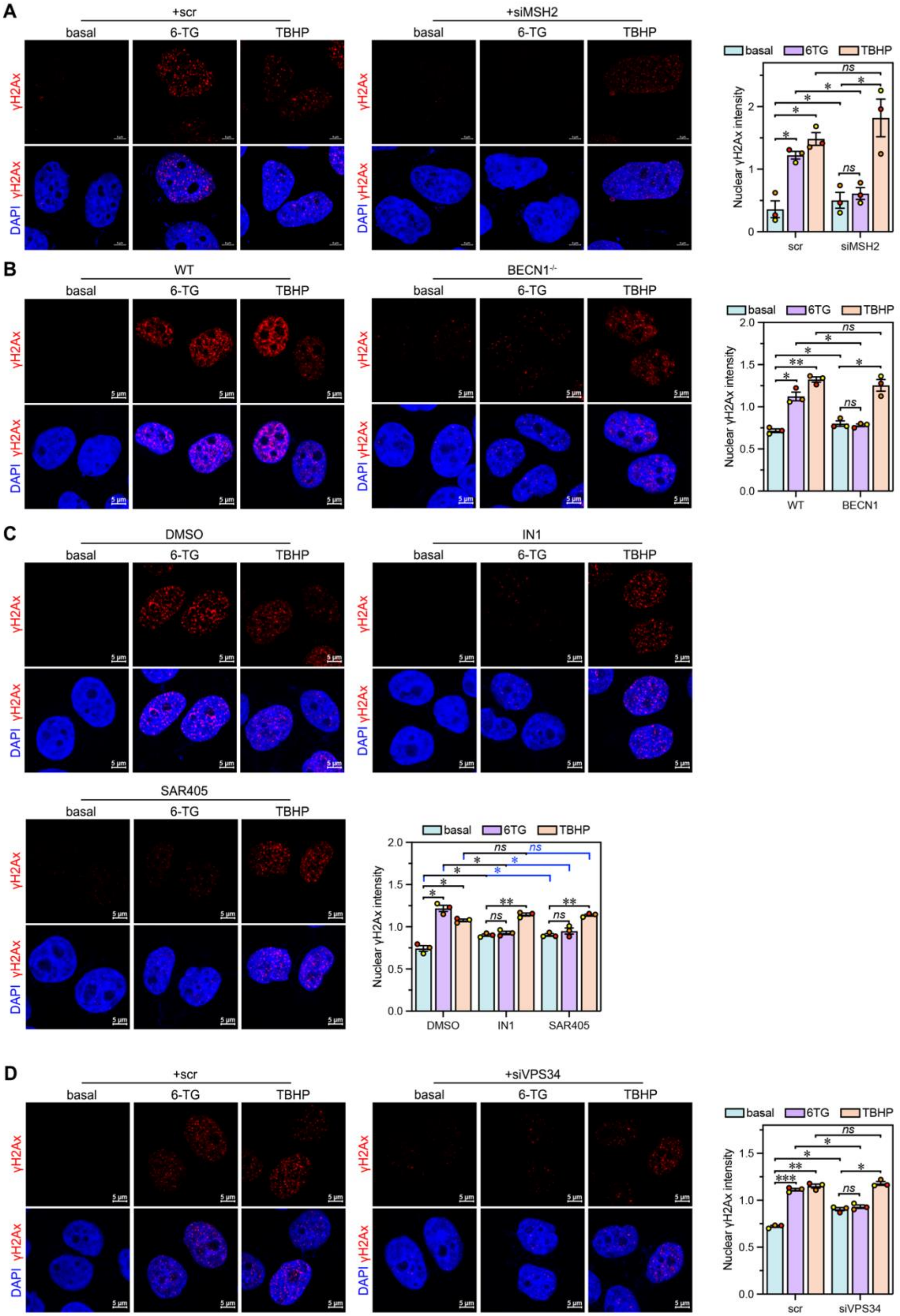
A
+scr
basal
6-TG
TBHP
+siMSH2
γH2Ax
DAPI γH2Ax
basal
6TG
TBHP
Nuclear γH2Ax intensity
ns
scr
siMSH2
B
WT
BECN1-/-
5 μm
Nuclear γH2Ax intensity
WT
BECN1
C
DMSO
IN1
SAR405
Nuclear γH2Ax intensity
DMSO
IN1
SAR405
D
+scr
+siVPS34
Nuclear γH2Ax intensity
scr
siVPS34

Supplementary Fig. 6: MMR-dependent γH2AX induction requires Beclin-1/Vps34

(A) Immunofluorescence analysis of nuclear γH2AX foci in HeLa cells transfected with scr or siMSH2, under basal conditions or following treatment with 6-TG (24 h after exposure to 30 μM 6-TG for 3 h) or TBHP (100 μM, 45 min). Scale bar: 5 μm. Quantification of nuclear γH2AX foci intensity is shown on the right. n = 3 biological replicates. $^{*}p < 0.05$.

(B) Immunofluorescence analysis of nuclear γH2AX foci in WT and $BECN1^{-/-}$ HeLa cells under basal conditions or following 6-TG or TBHP treatment. Scale bar: 5 μm. Quantification of nuclear γH2AX foci intensity is shown on the right. n = 3 biological replicates. $^{*}p < 0.05$, $^{**}p < 0.01$.

(C) Immunofluorescence analysis of nuclear γH2AX foci in HeLa cells treated with DMSO, IN1 (1 μM), or SAR405 (2 μM) for 16 h, under basal conditions or following 6-TG or TBHP exposure. Scale bar: 5 μm. Quantification of nuclear γH2AX foci intensity is shown on the right. n = 3 biological replicates. $^{*}p < 0.05$, $^{**}p < 0.01$.

(D) Immunofluorescence analysis of nuclear γH2AX foci in HeLa cells transfected with scr or siVPS34, under basal conditions or following 6-TG or TBHP treatment. n = 3 biological replicates. $^{*}p < 0.05$, $^{**}p < 0.01$, $^{***}p < 0.001$.

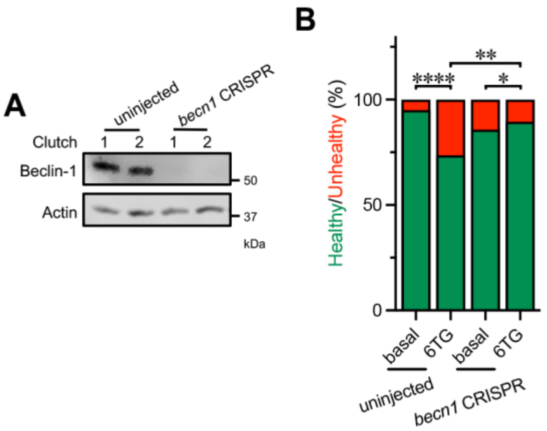

Supplementary Fig. 7: Beclin-1 deficiency confers 6-TG resistance in zebrafish

(A) Immunoblot confirming reduction of Beclin-1 protein in zebrafish injected with *becn1*-targeting CRISPR guides compared with uninjected controls.

(B) Toxicity assay to evaluate the genotoxic effect of 600 µM 6-TG in uninjected and becn1 CRISPR-injected zebrafish. Data is represented as the percentage of healthy *vs.* unhealthy zebrafish scored at 5 d.p.f. n=3 independent clutches per group. Statistical significance was assessed using Chi-square test. *$p < 0.05$, **$p < 0.01$, ****$p < 0.0001$.

Supplementary Table Legend

Raw data for mass spectrometry and relevant analysis.
